# An atlas of gene expression and gene co-regulation in the human retina

**DOI:** 10.1093/nar/gkw486

**Published:** 2016-05-27

**Authors:** Michele Pinelli, Annamaria Carissimo, Luisa Cutillo, Ching-Hung Lai, Margherita Mutarelli, Maria Nicoletta Moretti, Marwah Veer Singh, Marianthi Karali, Diego Carrella, Mariateresa Pizzo, Francesco Russo, Stefano Ferrari, Diego Ponzin, Claudia Angelini, Sandro Banfi, Diego di Bernardo

**Affiliations:** 1 Telethon Institute of Genetics and Medicine (TIGEM), Via Campi Flegrei 34, 80078 Pozzuoli, Italy; 2 Dipartimento Studi Aziendali e Quantitativi (DISAQ), Università degli studi di Napoli ‘Parthenope’, Via Generale Parisi, 80132 Napoli, Italy; 3 Istituto per le Applicazioni del Calcolo, Consiglio Nazionale delle Ricerca, Via Pietro Castellino 111, 80131 Napoli, Italy; 4 Fondazione Banca degli Occhi del Veneto, Via Paccagnella 11, 30174 Zelarino (Venice), Italy; 5 Medical Genetics, Department of Biochemistry, Biophysics and General Pathology, Second University of Naples, via Luigi De Crecchio 7, 80138 Naples (NA), Italy; 6 Dept. Of Chemical, Materials and Industrial Production Engineering, University of Naples ‘Federico II’, Piazzale Tecchio 80, 80125 Naples, Italy

## Abstract

The human retina is a specialized tissue involved in light stimulus transduction. Despite its unique biology, an accurate reference transcriptome is still missing. Here, we performed gene expression analysis (RNA-seq) of 50 retinal samples from non-visually impaired post-mortem donors. We identified novel transcripts with high confidence (Observed Transcriptome (ObsT)) and quantified the expression level of known transcripts (Reference Transcriptome (RefT)). The ObsT included 77 623 transcripts (23 960 genes) covering 137 Mb (35 Mb new transcribed genome). Most of the transcripts (92%) were multi-exonic: 81% with known isoforms, 16% with new isoforms and 3% belonging to new genes. The RefT included 13 792 genes across 94 521 known transcripts. Mitochondrial genes were among the most highly expressed, accounting for about 10% of the reads. Of all the protein-coding genes in Gencode, 65% are expressed in the retina. We exploited inter-individual variability in gene expression to infer a gene co-expression network and to identify genes specifically expressed in photoreceptor cells. We experimentally validated the photoreceptors localization of three genes in human retina that had not been previously reported. RNA-seq data and the gene co-expression network are available online (http://retina.tigem.it).

## INTRODUCTION

The retina is the specialized region of the central nervous system that transduces light stimuli into neural signals. It is formed by a concentric structure of three cell layers externally surrounded by the retinal pigment epithelium (RPE), composed of tightly connected cells that form the blood-retina barrier (1). The three retinal cell layers consist of different cell types with specialized functions. The retina is also the main target of a wide spectrum of disorders whose features depend on the cell type that is primarily affected (RetNet, https://sph.uth.edu/retnet/). Primary genetic defects of photoreceptor cells cause inherited retinal diseases (IRDs) that are characterized by broad clinical manifestations, ranging from isolated defects of the peripheral or central retina to reduced acuity in low-light condition to complete blindness (2). IRDs are the leading cause of blindness among the working adult population (2) and causative mutations have been found in over 100 genes (as for the RetNet website in January 2016). Several families affected by such conditions lack a conclusive genetic diagnosis (3–5) thus suggesting the presence of additional, yet to be identified, causative genes.

An accurate reference transcriptome (RefT) of the human retina may help in the discovery of new disease genes (6,7). This is especially true for a neural tissue such as the retina, where tissue-specific gene isoforms are more frequently found than in non-neural tissues (8,9). RNA-sequencing technology (RNA-seq) enables simultaneous assessment of both transcript structure and expression level within the same experiment (10). Only a few RNAseq studies have been performed in the human retina so far (11), with a limited sample size (maximum 8 individuals). Moreover, expression data from these earlier studies is not always publicly available. International efforts devoted to building RefTs in human tissues, such as the GTEx project (12), have largely neglected retina. The only exception is the Functional Annotation of the Mammalian Genome (FANTOM) project (13) where transcription start sites in a pooled sample of human retina were sequenced by means of the Cap Analysis of Gene Expression (CAGE) technology. The main reason for this lack of data is the difficulty in collecting high-quality human retina specimens.

Here, we collected 50 high-quality post-mortem human retinas from donors and performed high-coverage RNA-sequencing analysis to yield a comprehensive RefT of the human retina. Moreover, we exploited inter-individual variability in gene expression to infer a gene co-expression network and to predict, via a guilty-by-association approach, photoreceptor-specific expression of 253 genes. We experimentally confirmed this specific expression for three genes.

## MATERIALS AND METHODS

### Human retina sample collection

Retina samples were collected at Fondazione Banca degli Occhi del Veneto (FBOV) from 50 different donors for cornea transplantation in compliance with the tenets of the Declaration of Helsinki and after an informed consent allowing the use of tissues for research purposes was signed by the donor's next of kin (for a description of donors Supplementary Table S1). Each harvested tissue was accompanied with the FBOV progressive number and with details on the age and gender of donor, the cause of death and the total post-mortem time (T). To limit the possible effects of post-mortem time on RNA integrity and transcriptomic profiles, retinal tissues were isolated only from eye bulbs with a total post-mortem interval (T) ≤ 26 h. The average post-mortem time of the samples was 20.5 h (ranging from 6 to 26 h). For the same reason, bulbs deriving from multi-organ donors were excluded from the analysis. In order to limit cross-contamination with adjacent tissues, we established a protocol for the dissection of the retina from the eye bulbs (14). The dissected retinal tissue was visually inspected to exclude any cross-contamination with the pigmented RPE/choroid and was immediately submerged in RNA Stabilization Reagent (RNA later; QIAGEN).

### RNA extraction, library preparation and sequencing

Total RNA was extracted from the 50 human retina samples using the miRNeasy Kit (QIAGEN) according to the manufacturer's instructions. RNA was quantified using a NanoDrop ND-8000 spectrophotometer (NanoDrop Technologies) and the integrity was evaluated using an RNA 6000 Nano chip on a Bioanalyzer (Agilent Technologies). The RNA of the 50 samples had an average RNA integrity number (RIN) of 8.7 (ranging from 7.2 to 9.7). Libraries were prepared according to manufacturer's instructions (TruSeq RNA Sample Preparation kit) with an initial amount of 4 μg of total RNA. Quality control of library templates was performed using a High Sensitivity DNA Assay kit (Agilent Technologies) on a Bioanalyzer (Agilent Technologies). Qubit quantification platform was used to normalize samples for the library preparation (Qubit 2.0 Fluorometer, Life Technologies). Libraries were sequenced via a paired-end chemistry on an Illumina HiSeq1000 platform with an average yield of ∼6 Mb.

### Data analysis

**Figure 1.**
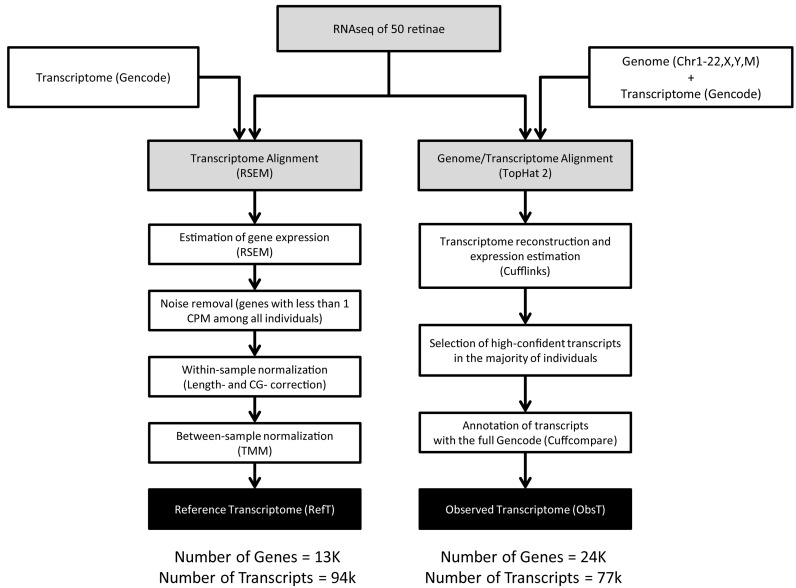
The bioinformatics pipeline to analyse the 50 RNA-seq samples obtained from human donors.

The exploratory analysis, whose steps are shown in Figure 1, was carried out with the ‘tuxedo’ software suite (Trapnell *et al*., 2010) and led to the definition of the Observed Transcriptome (ObsT). The conservative analysis (Figure 1) was carried out by running the RNA-Seq by Expectation-Maximization (RSEM) package (Li & Dewey, 2011) and led to the definition of the RefT.

### Reference transcriptome

Trimmed reads were aligned to Gencode transcripts by running RSEM with standard parameters (15). Aligned reads (counts) were then used to estimate the gene expression level of each Gencode transcript. Only genes with at least 1 count per million reads (1 CPM) in each of the 50 samples were considered as expressed. By removing low count reads, the histogram of count frequency versus logged length approached a Gaussian-like distribution (data not shown). Gene expression values were then normalized within and between samples. The within-sample normalization corrects for gene-specific effects, e.g. related to gene length or GC-content. Visual inspection of the data by scatter plots revealed a nonlinear trend between count and gene length but no trend with GC-content was apparent (Supplementary Figure S1). We thus adopted a gene length normalization method, namely full-quantile normalization as discussed in Risso *et al*. using the *withinLaneNormalization* function from the Bioconductor package EDASeq. Between-sample normalization is needed to correct for differences such as the sequencing depth. To this end, we employed the TMM (trimmed mean of M values) normalization procedure (16,17) (Supplementary Figure S2). Finally, we performed GSEA (18) with the ‘Gene Ontology’ gene-set (MSIG ver 5) for functional interpretation of the most expressed genes.

### Observed transcriptome

Trimmed reads were aligned to the human genome, version hg19 as downloaded from UCSC genome browser website (19), by using TopHat2 (20). TopHat2 allows RNA-seq alignment guided by a RefT structure. To this end, we used as reference a curated version of Gencode containing only high confidence (level 1 and 2), mitochondrial and non-redundant transcripts. According to this procedure, the reads were preferentially aligned to the Gencode transcripts and, when not possible, to the rest of the genome. In order to compute the average genome-wide coverage (AC track) of the retina transcriptome, the human genome (hg19) was divided into bins of 100 nucleotides and then read coverage was calculated in each bin by summing up the contribution of all the 50 samples. Bins covered by less 100 reads were considered as not expressed and assigned a ‘bin coverage’ value of 0. For bins with more than 100 reads, a ‘bin coverage’ value was computed by first dividing the number of reads falling in the bin in each sample by the total number of aligned reads in that sample and then by averaging across the 50 samples. The ObsT was inferred by using Cufflinks (20) guided by the Gencode RefT structure (the same one used for the alignment). Cufflinks tries to resolve the reads into the known transcripts whenever possible and to infer novel transcripts otherwise. Transcripts were first inferred at a single sample level. The 50 sample-level predictions were then merged into a non-redundant consensus using cuffmerge and cuffcompare and all the transcripts that had a 95% confidence interval including 0 in more than 50% of male or female samples were removed. This filtering corresponds to retain in the analysis only the genes that have a probability of 0.5 of being present in one sample according to the binomial distribution. We then applied cuffcompare to annotate ObsT transcripts overlapping with Gencode transcripts. With very few exceptions, all of the ObsT transcripts had some overlap with Gencode transcripts. To check whether the hypothetical encoded proteins were also similar between ObsT and Gencode transcripts, we compared the predicted open reading frame (ORF) of ObsT transcripts with the Gencode transcripts they overlapped with. The ORF prediction was performed by selecting the longest ORF with a starting and a stop codon within the transcript. The bedtools ver 2.23, python ver 2.6.6, ver R 3.1.1 were used for the bioinformatic and statistical analysis.

### Gene network

The co-regulated genes were identified by computing the Spearman correlation coefficient (SCC) between pair of genes across the 50 samples. To this end, we quantified the raw counts for each gene with RSEM and we then applied the TMM between-sample normalization, but no within-sample normalisation was applied, since the SCC is unaffected by it. Only genes with at least 1 CPM per sample were included in the analysis. For each gene we also excluded outliers using the conventional upper and lower limits of Q3 + 1.5 × (Q3 − Q1) and Q1 − 1.5 × (Q3 − Q1), respectively, keeping only samples within this region. A threshold for the SCC of 0.85 was used to identify significantly co-expressed genes. This threshold value corresponds to select 1.5% of all the possible gene-pairs that can be formed by the 13.8K genes. In order to assess whether the gene network captures known functional interactions among genes, we used as a ‘gold-standard’ network, the collection of physical and functional interactions between genes reported in the Search Tool for Recurring Instances of Neighbouring Genes (STRING) database (21). In STRING, each interaction is associated with a score ranging from 0 to 999. A score ≥700 is recommended by the STRING authors to call high confidence interactions. Hence, we selected interactions with a score above 700 as the gold-standard network. We then ranked gene-pairs in our gene co-expression network according to their unsigned SCC in descending order and computed the receiver operating characteristic (ROC) curve by comparison to the STRING gold-standard network. The resulting ROC is shown in Supplementary Figure S3 together with the ‘random’ ROC obtained by ranking gene-pairs randomly.

### Guilty-by-association

In order to identify genes that are expressed specifically in photoreceptor cells, we applied a guilty-by-association strategy to the co-expression network, as previously described (22). Briefly, we first selected a list of known photoreceptor-specific genes (PG, Supplementary Table S2) from the literature, which was not meant to be exhaustive. Next, for each gene in the network, we checked whether the genes connected to it (i.e. its neighbours) were enriched for PG. A gene was considered a candidate photoreceptor gene (CPG) when it had among its neighbours a number of PG larger than the expected one, according to (the False Discovery Rate (FDR)-corrected) Fisher's exact test. If the number of gene neighbours was larger than 200, only the top-200 most correlated genes were retained for the guilty-by-association analysis. Similarly, in order to identify candidate genes for retinal diseases, we compiled a list of genes known to cause retinal disease when mutated (RetNet disease gene (RDG), Supplementary Table S2) from the RetNet website (accessed January 2015). The list of RDG was used as input for the guilty-by-association analysis, thus obtaining a list of candidate disease genes (CDG).

### RNA *in situ* hybridization

RNA *in situ* hybridization assays were performed on cryo-sections. Antisense probes and sense (control) probes for the tested transcripts were generated by polymerase chain reaction (PCR) on human genomic DNA using primers that were tailed with sequences recognized by the RNA polymerases T3 and T7 (Supplementary Table S3). PCR products were purified and used as templates for *in vitro* cRNA transcription. To detect the probe, a hapten tag (digoxigenin-labeled Uridine-5′-triphosphate (UTP)) was incorporated into the RNA during the *in vitro* transcription reaction (DIG RNA labelling kit; Roche). RNA ISH experiments on human eye sections using cRNA probes were performed as previously described (23). Hybridization was performed at 65°C.

### Disease variants from Human Gene Mutation Database (HGMD)

Disease variants involved in different diseases were extracted from HGMD (ver 2015) by applying broad filters. The ‘ocular’ disease mutation group was extracted by searching for the following text string in the phenotype description field: ‘retina’, ‘eye’,’ vision’, ‘visual’, ‘blind’. Similarly, (i) cardiac-, (ii) liver-, and (iii) immunological-disease groups were extracted by using corresponding keywords: (i) ‘cardiac’, ‘heart’, (ii) ‘liver’, ‘hepatic’ and (iii) ‘immune’. A further requirement was that variants had to be ‘causative’ and not just altering the disease risk.

## RESULTS

We collected post-mortem tissue samples from 50 donors not affected by retinal diseases (Table 1 and Supplementary Table S1). RNA sequencing was performed on all of the samples, as detailed in the Materials and Methods section, and it generated a total of 3550 M reads (average 72 M reads per sample, range 40–110 M) corresponding to 290 260 Mb of sequence (average 5924 Mb per sample, range 2926–11 045 Mb). On average 79% of the reads were successfully mapped to the human genome reference version hg19 (Supplementary Figure S4 and Table S4).

**Table 1.** Donor characteristics and average RNA integrity number (RIN)

Gender	18 Female and 32 Male donors
Average Age	61.4 years (range 42÷72)
Cause of death	Neoplastic disease: 38
	Cardiovascular disease: 7
	Injury/poisoning: 3
	Digestive disease: 2
Average Sample Collection Time (from time of death)	20.5 h
Average RIN value	8.5

We generated a UCSC genome browser track to report the average coverage (AC) across the genomic sequence by counting the number of mapped reads per 100 bp averaged across the 50 samples. The final length of genome that was covered by mapped reads was 238 Mb, corresponding to 7.78% of the genome (http://retina.tigem.it - ‘Browser’ tab). Only a part of this region (∼178 Mb) was already reported to be expressed according to the Gencode RefT (Supplementary Figure S5). The median coverage was of 376 reads/100 bp (∼4 reads per bp) (Inter Quartile Range (IQR) = 173 ÷ 1296 reads/100 bp). Interestingly, the median coverage was much higher in the mitochondrial genome with 2 215 966 reads/100 bp (∼22 160 reads per bp) (IQR = 2 215 966 ÷ 4 920 964 reads/100 bp), which is surprisingly high even considering the presence of thousands of mitochondria per cell.

RNA-seq data were analysed following the pipeline described in Figure 1. Specifically, we performed: (i) a reference-based *de novo* transcriptome reconstruction in order to identify putative novel transcripts expressed in the human retina (ObsT) and (ii) a RefT analysis, in order to estimate the level of expression of known genes in the human retina (RefT). Both analyses benefited from the availability of 50 different samples, which were used to increase sensitivity and specificity in the estimation of novel transcripts and to increase the precision in the estimation of the expression level of individual transcripts. The bioinformatic analysis pipelines to obtain the ObsT and the RefT differ mainly in the read mapping strategy. The RefT pipeline employs RSEM, a reference-based strategy to estimate gene expression levels starting from a set of reference transcript sequences. The ObsT pipeline makes use of Tophat2 and Cufflinks, which enable the discovery of novel transcripts at the cost of a loss of precision in the estimation of the expression level of known transcripts.

### Obst

**Figure 2.**
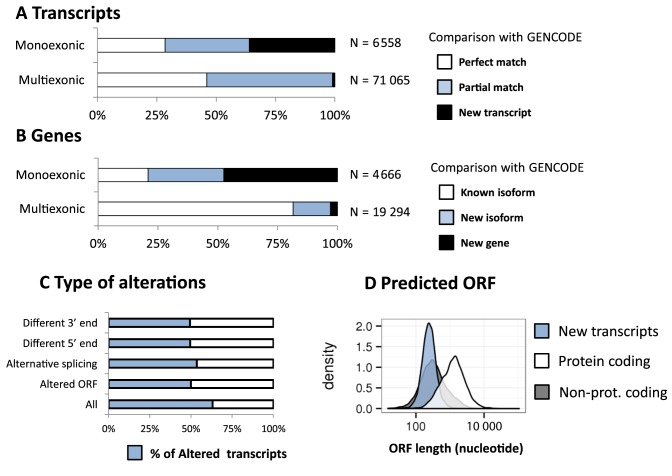
Overview of the Observed Transcriptome (ObsT). (**A**) Distribution of ObsT monoexonic transcripts and multiexonic transcripts that either perfectly overlap a Gencode transcript (white), or share at-least one exon–exon junction with a Gencode transcript (blue), or share no exon–exon junctions with Gencode transcript (black). (**B**) as in (A) but using genes rather than transcripts. (**C**) The frequency of transcripts’ alterations found in the ObsT compared to the known reference sequence in Gencode. (**D**) The predicted ORF length of known-protein-coding transcripts (white), known non-protein-coding transcripts (grey) and new transcripts found in the ObsT (blue); y-axis: the transcript density over the ORF-length; x-axis: ORF-length on a log-10 scale.

The ObsT was reconstructed by running TopHat 2 and Cufflinks on the sequencing data for each of the 50 samples (Materials and Methods). In order to find a balance between a precise detection of rare transcripts and a small number of False Positives, we performed an ensemble analysis of the 50 samples, as follows. We first run *cufflinks* to infer the per-sample transcript structure from the TopHat2 aligned reads. An expression level and a 95% confidence interval were assigned to each transcript. To merge the transcriptome of the single samples into a unique RefT, we removed transcripts whose expression confidence interval included 0 and only retained those transcripts present in the majority of both male and female individuals. We thus identified 282 980 different exons that were joined into 77 623 isoforms for 23 960 genes (Figure 2A, B and Supplementary Table S5). The ObsT covered 137 Mb (4.5%) of the reference genome sequence, 35 Mb of which were not annotated as transcribed regions in Gencode (Supplementary Figure S5). About 8% (6558) of the transcripts were single-exon that generally belonged to single-isoform genes. About half of them overlapped with a known transcript, and a third of them with perfect identity (Figure 2A). Of the 2366 novel single-exon transcripts, 328 (13%) overlap with at least one lncRNA according to the database by Cabili *et al*. (2011). Multi-exonic transcripts (71 065) were found for 19 294 genes (Figure 2A, B and Supplementary Table S5). Of these genes, 81% had at least one isoform whose exon–exon junctions perfectly matched a known Gencode transcript. The remaining isoforms were either putative new alternative isoforms (16%) of known genes or entirely new transcripts (3%).

Most of these new transcripts were likely non-coding as the predicted length of the ORF was much shorter than those of known protein-coding genes (Figure 2D). Sixty of these new transcripts (belonging to 41 genes) mapped to non-standard genome contigs (i.e. ‘unassembled’ or ‘alternative’) and 53 of them overlapped a transcribed region (according to the UCSC gene or the spliced-EST track) (Supplementary Figure S6 reports two examples). Since the ObsT was reconstructed with the guidance of a reference genome that included only the standard chromosomes (1-22, X, Y, mitochondrial), these 53 transcripts were predicted *de novo* by the bioinformatics analysis pipeline, thus confirming the reliability of the ObsT. Hereafter, we will not further consider genes and transcripts mapped to non-standard chromosomes.

About 63% of the ObsT transcripts showed some differences compared to the most similar Gencode transcript. The most frequent alteration was the presence of at least one different alternative splicing event (Figure 2C). The predicted protein sequence of half of the transcripts (i.e. the longest ORF) was also altered. Interestingly, the most expressed isoforms were also the least variable (Supplementary Table S6, Figure S7). Finally, transcripts with a perfect overlap with Gencode were more expressed than those with a partial overlap and, in turn, those with a partial overlap were more expressed than those with no overlap (new transcripts) (Supplementary Figure S8).

### Reft

The RefT was generated as shown in Figure 1. Briefly, for each of the 50 samples, RSEM was used to estimate the raw counts of the transcripts annotated in Gencode. The raw counts were normalized intra-samples and inter-samples following standard procedures (Materials and Methods) (16,17,24). Next, in order to obtain a single value of expression for each gene from the 50 samples, the median normalised count was computed. The final RefT included 13 792 genes across 94 521 transcripts (Supplementary Table S7A and Table S7B).

**Figure 3.**
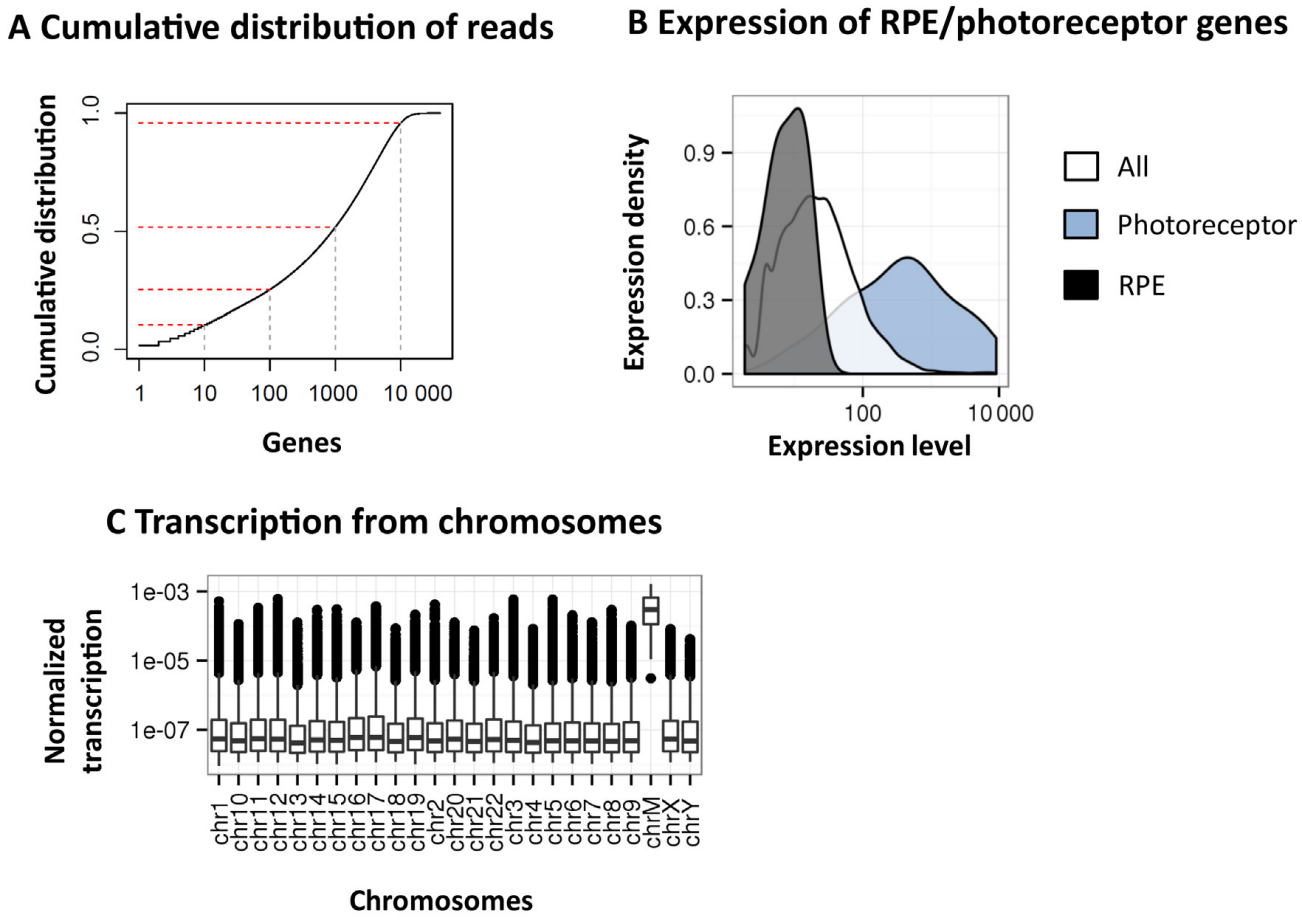
Overview of the Reference Transcriptome (RefT). (**A**) Cumulative distribution of mapped reads on gene regions prior to normalisation and filtering. y-axis: cumulative distribution of reads; x-axis: the number of genes sorted by coverage in descending order (log10). Top-1, top-10, top-100, top-1000 and top-10K expressed genes are marked. (**B**) Density distribution of expression for the genes in Gencode (white), for the photoreceptor-specific genes (blue), and for RPE-specific genes (black). Gene density (y-axis) over the normalised expression level (x-axis) on a log-10 scale is shown. (**C**) The average expression of genes in the human retina divided by chromosome.

Principal component analysis (PCA) was used to check for potential biases across the 50 samples. No obvious clustering was apparent in the PCA plots (Supplementary Figure S9A) except when using sex chromosomes alone, where two distinct male and female clusters appear as expected (Supplementary Figure S9B). PCA analysis thus confirms that sequencing was not flawed by potential confounding factors either related to the experimental procedure (collection of biological samples, post-mortem time, reagent lots) or to donor characteristics (age and cause of death). Similarly, contamination with RPE cells was excluded by comparing the expression of RPE-specific genes (25) with the overall gene expression and with that of photoreceptor-specific genes (Figure 3B and Supplementary Figure S10).

The top-1000 most expressed genes in the human retina are covered by 50% of the reads, whereas the top-10 000 by almost 95% (Figure 3A). Genes localised to the mitochondrial chromosome (e.g. MT-ATP6, MT-CYB, MT-ND4, MT-CO1) were among the most highly expressed genes, accounting for about 10% of the total reads (Supplementary Figure S11). Out of all the genes annotated in Gencode, 23.8% are expressed in the retina, with a larger proportion of genes annotated as ‘protein-coding’ (65%, Supplementary Table S7).

Overall, highly expressed genes were enriched by functional classes related to visual perception (Supplementary Table S8 and Figure S12). Retinal disease-genes and photoreceptor-specific genes were also highly expressed (Supplementary Table S9 and Figure S13). Genes highly variable among individuals (high Coefficient of Variation, (CV)) were enriched for functional classes related to the extracellular matrix and transmembrane ion permeability (Supplementary Table S10A and Figure S14A). On the contrary, the least variable genes were enriched for classes related to the housekeeping functions (Supplementary Table S10B and Figure S14B).

The ObsT and RefT share 13 367 genes in common, corresponding to about half (56%) of ObsT genes and almost all (96%) of the RefT genes (Supplementary Figure S15A). The expression levels of these shared genes were similar in both the ObsT and the RefT (Supplementary Figure S15B, SCC *P*-value < 10^−16^). Although the number of genes was much higher in the ObsT than in the RefT, only a minority (19%) of the ObsT transcripts belonged to genes without any isoform expressed in RefT (data not shown). In addition, ObsT-only genes were generally less expressed (SF, Kruskal–Wallis test *P*-value < 10^−16^, Supplementary Figure S16) than those expressed also in the RefT and were composed by a smaller number of transcripts (median 4 versus 1).

### Gene Co-expression network and identification of novel candidate photoreceptor genes

We hypothesised that inter-individual variability in gene expression could be exploited to identify co-expressed genes in the human retina. Gene co-expression implies functional or physical interactions among genes (26,27). We used the SCC to quantify gene–gene co-expression for all of the gene-pairs in the RefT across the 50 samples. Since the RefT includes 13 792 genes, we computed the SCC for 95 102 736 gene-pairs. In order to have a manageable number of gene-pairs to work with, we selected a threshold for the SCC equal to 0.85, and selected only gene-pairs with an SCC higher than 0.85. The resulting gene network consists of 11 022 genes and 1 401 990 edges connecting gene-pairs, which correspond to about 1.5% of all the possible gene-pairs. About one-half of the genes (53%) had more than 100 edges. In order to assess the biological significance of the gene co-expression network, we used as the *gold standard* a collection of functional and physical gene–gene interactions reported in the STRING database (28). We sorted gene-pairs in the network according to their absolute SCC value in descending order and then checked whether each gene-pair was supported by STRING or not. As shown in Supplementary Figures S3A and B, we estimated the percentage of correct connections to be significantly higher had these been randomly guessed. These results confirm that the gene network, which we inferred by exploiting inter-individual variability in gene expression, contains some biologically relevant information.

**Figure 4.**
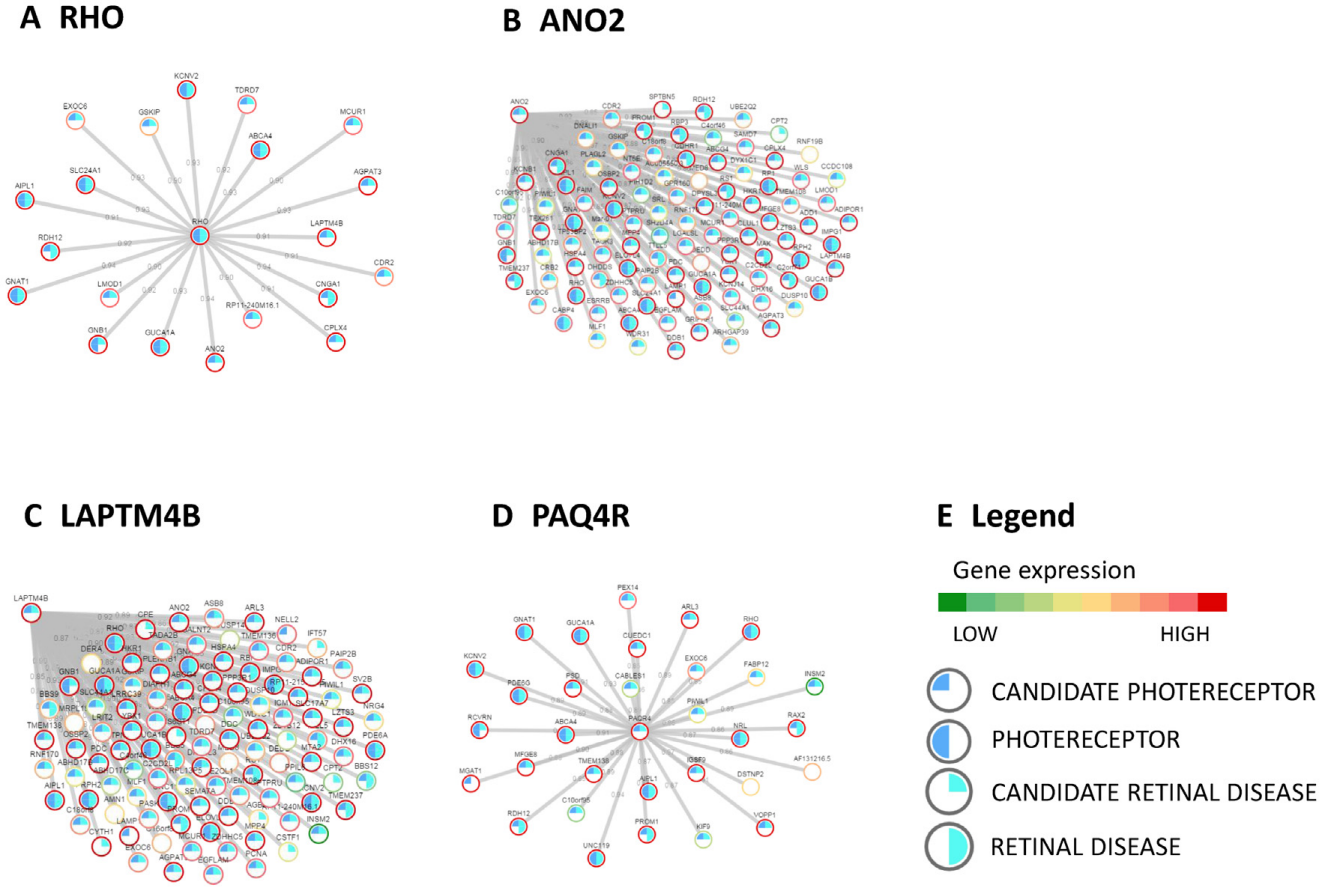
Gene co-expression network and guilty-by-association analysis. (**A**) The genes co-expressed with RHO, one of the most expressed and functionally important retina genes, according to the gene co-expression network inferred from the 50 retina samples. (**B**), (**C**) and (**D**) show the genes co-expressed with three Candidate Photoreceptor Genes (CPG): ANO2, LAPTM4B and PAQ4R. Grey lines represent gene-correlations higher than 0.85; ring colour represents gene expression and filling colour reports the gene being Photoreceptor gene, CPG, Retinal disease gene and Candidate retinal disease gene. (**E**) The legend for gene networks in Panel A, B, C and D.

Gene networks can be used to assess the function or tissue-specific expression of a gene via a *guilty-by-association* approach (22,29). This approach consists in assigning a function to a gene by checking whether there is a shared function among its ‘gene neighbours’, i.e. the set of genes connected to it in the network. We used the guilty-by-association approach to identify genes specifically expressed in photoreceptors cells. To this end, we first compiled a list of 60 genes known to be specifically expressed in photoreceptors (PG list in Supplementary Table S2), we then searched for genes in the network whose gene neighbours were significantly enriched in the PG list. This guilty-by-association analysis resulted in 253 candidate photoreceptor-specific genes (CPG, Supplementary Table S11) ranked according to the significance of their enrichment. Thirty-four CPGs were included in the original PG list (Odds ratio = 110, Fisher's *P*-value < 10^−16^, Supplementary Table S12), thus confirming the validity of our approach. For example, Figure 4A shows that most of the genes co-expressed with *RHO* are indeed PG like *RHO* itself. Table 2 shows the top-15 most significant CPGs (Supplementary Table S11 reports the whole list). Interestingly, for most of these genes, we found literature and database data supporting their expression in photoreceptor cells (Supplementary Text).

**Table 2.** Candidate photoreceptor genes (CPGs) resulting from the guilty-by-association analysis of the gene co-expression network

Gene	FDR	Expr. rank	Comment	Neighbour GO Enrichment
DPYSL3	6.25E-21	184	Neurite and axonal outgrowth	Visual perception
				Photoreceptor outer segment
				Photoreceptor activity
C2orf71	4.05E-19	1041	Retinitis Pigmentosa 54	Fatty acid elongation, polyunsaturated f…
				Calcineurin complex
				Fatty acid elongase activity
CPLX4	8.37E-18	380	Expressed in mouse rod cells	Chaperone-mediated protein complex assem…
				Phagolysosome membrane
				Lipid transporter activity
GSKIP	1.16E-17	4593	Potentially involved in photoreceptor cell survival	Visual perception
				Photoreceptor disc membrane
				3′,5′-cyclic-nucleotide phosphodiesteras…
ANO2*	1.42E-16	320	Potentially involved in photoreceptor membrane potential and firing	Detection of light stimulus
				Photoreceptor outer segment
				Potassium ion transmembrane transporter …
WLS	2.83E-16	1902	Mediate many developmental processes during embryogenesis	Detection of light stimulus
				Photoreceptor outer segment membrane
				Voltage-gated potassium channel activity
GPR160	7.59E-16	2794	G-protein coupled receptor with unknown function	Visual perception
				Nonmotile primary cilium
				Mannosyl-oligosaccharide 1,3-1,6-alpha-m…
PAIP2B	1.63E-15	3730	Influence membrane potential and light-dependent firing	Visual perception
				Cilium
				Calmodulin binding
TMEM136	2.05E-15	2088	Transmembrane protein is expressed in mouse neuroretina	Cellular potassium ion transport
				Calcineurin complex
				Voltage-gated potassium channel activity
AGPAT3	3.47E-15	243	Ubiquitous acetyltransferase involved in lipid metabolism	Visual perception
				Photoreceptor outer segment
				Voltage-gated potassium channel activity
PAQR4*	3.89E-15	673	Structurally similar to ADIPOR1 and ADIPOR2	Visual perception
				Photoreceptor disc membrane
				3′,5′-cyclic-gmp phosphodiesterase activ…
LMOD1	6.39E-15	1440	Muscular antigen involved in Thyroid Ophthalmopathy	Visual perception
				Photoreceptor outer segment
				Biotinidase activity
MEF2C	7.03E-15	5685	TF involved in multi-organs development including brain	Visual perception
				Cilium
				Calmodulin binding
LAPTM4B*	1.49E-14	299	Lysosome-associated protein expressed in many tissues	Visual perception
				Cilium
				Retinal binding

Only genes ranked in the top 15 positions according to their significance in the guilty-by-association analysis are shown (for the full list refer to Supplementary Table S13). FDR: false discovery rate. Expr. Rank: rank of the gene in the retina with respect to its expression level (e.g. 1 being the most expressed). Comment: a manual-curated comment obtained by querying OMIM, PubMed and MGI. Neighbour GO Enrichment: the most enriched GO term among the genes’ neighbours in the co-expression network.

**Figure 5.**
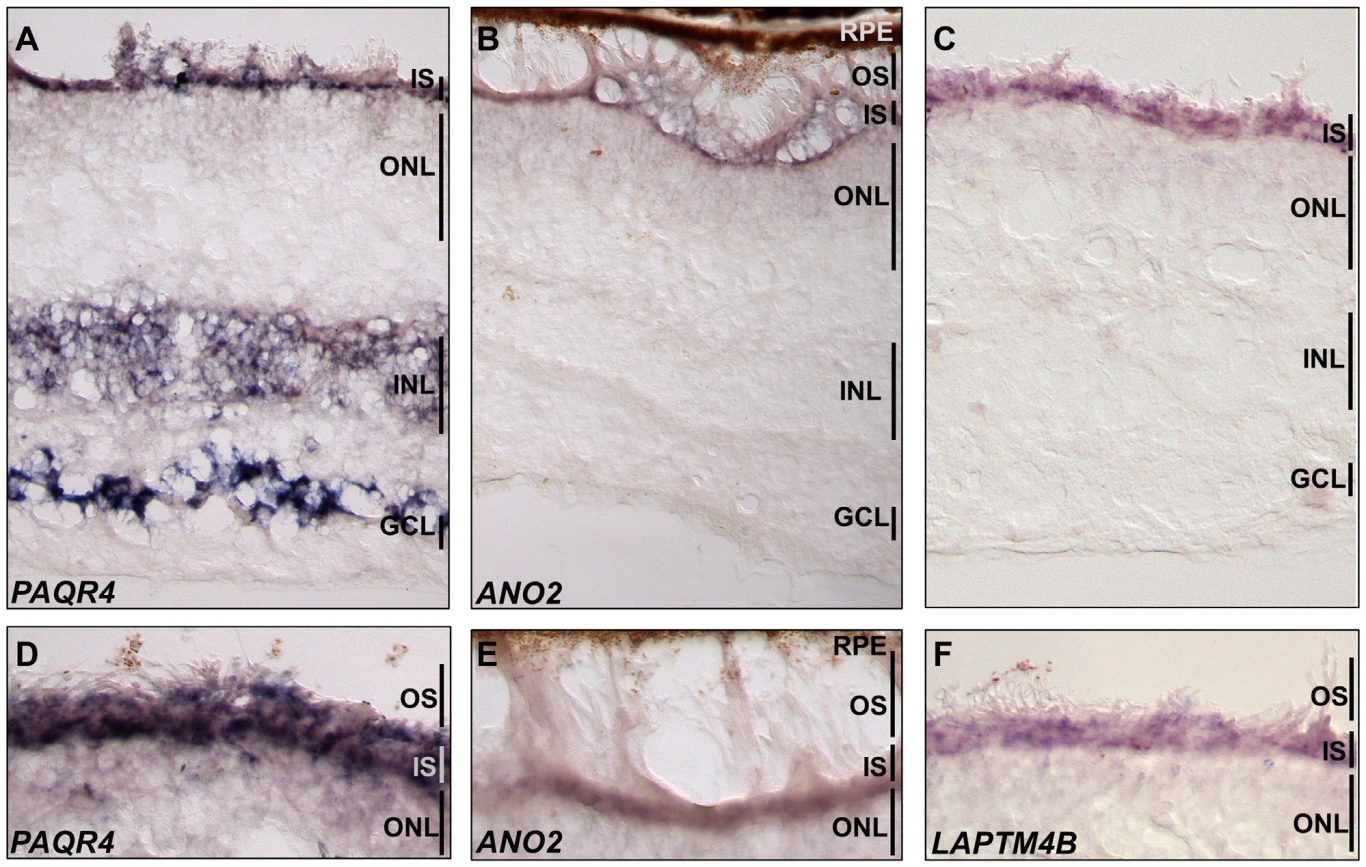
*In situ* hybridisation in human retina for three candidate photoreceptor genes. Hybridisation of human retina sections with cRNA probes for (**A** and **D**) PAQR4, (**B** and **E**) ANO2 and (**C** and **F**) LAPTM4B. For all three transcripts, a strong staining is detected in the inner segments of photoreceptors (IS). PAQR4 is also expressed in the INL and strongly in the GCL. Figures D, E and F show a higher magnification of the photoreceptor inner and outer segments. Ganglion Cell Layer; INL, Inner Nuclear Layer; IS, Photoreceptor Inner Segments; ONL, Outer Nuclear Layer; OS, Photoreceptor Outer Segments; RPE.

We selected three genes from Table 2 for experimental validation by means of RNA *in situ* hybridization on human retina tissues (*ANO2, PAQR4, LAPTM4B)*. These genes have not been previously reported to be expressed in photoreceptor cells and limited knowledge of their roles in retina pathophysiology is available in the literature: *ANO2* (Figure 4B), whose mouse orthologue is known to be expressed in mouse retina, is involved in the regulation of cellular membrane electric potential (30); *PAQR4* (Figure 4C) is a paralog of adiponectin receptor 1 and 2 (*ADIPOR1* and *ADIPOR2*), both known to be expressed in photoreceptor cells. In addition *ADIPOR1* knock-out causes a form of photoreceptor degeneration in mouse (31); *LAPTM4B* (Figure 4D) encodes for a lysosome-associated transmembrane protein that has been never implicated in retina physiology. RNA *in situ* hybridization experiments confirmed that all of the 3 genes are significantly expressed in the photoreceptor layer (Figure 5).

Gene networks have also been used in the literature to prioritise disease-associated genes and for the diagnosis of rare genetic disorders (32). We, therefore, decided to apply the guilty-by-association approach to identify candidate retinal disease genes (CDG, Supplementary Table S13). To this end, we first compiled a list of known retinal disease genes (RDG) from the RetNet database (Supplementary Table S2). We then searched for genes in the network whose gene neighbours were significantly enriched in the RDG list. As in the case of photoreceptor genes, there was a significant enrichment of known RDG among the CDG (Odds ratio = 15, Fisher's *P*-value < 10^−16^, Supplementary Table S12). Moreover, since we first compiled the list of RDG (January 2015), 10 new genes have been found to cause retinal diseases according to the RetNet database (*ATF6, DRAM2, HGSNAT, IFT172, LAMA1, NEUROD1, POC1B, PRPS1, SPP2, KIZ*). One of them (*IFT172*) was among our CDG (enrichment *P* = 0.046). We thus believe that the CDG list may be useful to researchers and clinicians and hence, we made it available online (http://retina.tigem.it).

## DISCUSSION

We performed RNA-seq based transcriptome analysis of 50 retina samples from non-visually impaired post-mortem donors. We estimated gene expression levels for all the known genes and derived a gene co-expression network exploiting inter-individual variability in gene expression. We identified a list of genes potentially expressed in photoreceptor cells and a list of candidate disease genes involved in retinopathies. We also inferred novel retina-specific isoforms by *de novo* alignment of the RNA-seq reads to the whole genome.

We applied two different bioinformatics pipelines to analyse the RNA-seq data, one to obtain a high-confidence RefT and the other to maximise the possibility of finding novel transcripts (ObsT) (Figure 1). This dual strategy enabled both a precise estimation of the expression of all known genes and an overview of potentially novel transcripts.

Interestingly, a large fraction of observed transcripts originated from the mitochondrial chromosome (Figure 3C and Supplementary Figure S11). This phenomenon has been observed also in other tissues in the GTex study (12) and it is in agreement with a previous study on retina transcriptome (33). Mitochondrial gene overexpression in the retina is probably related to the high energy demand of this tissue (34). Indeed, mutations in mitochondrial genes are known to cause retinal diseases (RetNet). Furthermore, we found that genes with the ‘mitochondrial’ Gene Ontology (GO) localization, either encoded by the mitochondrial or nuclear genome, were more expressed than other genes (Mann–Whitney U-test *P* < 10E-16, data not shown). We excluded a potential contamination with mitochondrial genome by observing that the read coverage in exonic regions of mitochondrial genes was much higher than that in non-exonic regions (Supplementary Figure S17).

RNA-seq data yield information both on isoform structure and expression in a single experiment, however, automatic transcript reconstruction is not always reliable. It is known that *de novo* transcriptome assembly performs well in the identification of regions that are actively transcribed but tends to inaccurately define the 5′ and 3′ endpoints and exon–exon junctions (35). We found that most of the inferred retina transcripts had some evidence of alteration compared to the reference sequence in Gencode (59.1%, Figure 2C, Supplementary Table S6). This is probably an overestimation due to our decision to increase the ability to discover new transcripts at the cost of an increase in the False Positive rate. Nevertheless, our transcript reconstruction pipeline is able to keep the False Positive rate low by using the 50 samples to filter out unreliable sample-specific transcripts. Indeed, we were able to correctly reconstruct transcripts in genomic regions that were not supplied for the ‘guided’ alignment and transcript-inference (Supplementary Figure S6).

The availability of 50 samples allowed us to correlate gene expression among samples and to infer a gene co-regulation network, which, despite its limitations, may be useful in elucidating gene function. We confirmed that genes, which are known to be expressed in specific cells or involved in specific functions, tended to be co-expressed and exploited this information to find candidate photoreceptor genes. Similarly, we inferred a list of candidate disease genes with a potential pathological role. The most significant candidate disease gene, *GNB1*, was already investigated as a potential disease-gene on the basis of biomedical literature (36). It was proven to cause a form of retinitis pigmentosa in a murine model (37,38), but its definitive involvement in human disease is still uncertain. The second most significant candidate disease gene was *PAIP2B*, which was expressed at an intermediate level (rank 3730) and was predicted also to be a candidate photoreceptor gene (*P* = 10^−15^). Interestingly, this gene inhibits the translation of mature mRNAs by displacing PABP from the 3′-end (39). If the involvement of this gene in the retinal pathophysiology will be confirmed, it supports the idea that the retina has a low tolerance for dysregulation in RNA-maturation, in addition to its known sensitivity to splicing machinery dysfunction (8). Interestingly five of the candidate disease genes mapped to 3 genomic regions linked to retinopathies for which the causative genes are still unknown (RetNet). *PHLPP2, KIFC3, CCDC113* localized within the Optic Atrophy 8 locus (OPA8, OMIM 616648) that was linked (LOD score 8.8) to a familiar form of optic neuropathy (40); *NT5E* mapped within the Retinitis Pigmentosa 63 locus (RP63, OMIM 614494) linked to a dominant form of retinitis pigmentosa (41) and *TTC40* to a dominant form of cone-rod dystrophy (CORD17) (42).

The retina transcriptome can be used for the prioritization of variants obtained from a sequencing study with diagnostic/mutation-discovery purpose. Indeed, it is known that selecting a representative transcriptome impacts the prioritization efficacy (43) and this must be especially true for tissues like retina, which makes a large usage of organ-specific transcripts (8,9).

To date, other two RNA-seq analyses of the human retina transcriptome have been reported in the literature: Farkas *et al*. (33) analysed 3 samples, while Li *et al*. (44) analysed 8 samples. Farkas *et al*. were mainly interested in the description of the neuroretina transcriptome, whereas Li *et al*. performed differential expression analysis between neuroretina and pigment epithelium and between macula and periphery. Compared to these studies, ours benefited from a larger sample size enabling a more precise estimation of gene expression and identification of alternative splicing events and novel transcripts. Farkas *et al*. performed a *de novo* transcript reconstruction analysis on three samples and they identified about 160K transcripts (compared to our 94K – ObsT), with 29 887 novel exons, 28 271 exon skipping and 21 757 alternative 5′ and 3′ exon events. Despite the difference in the total number of novel transcripts, both Farksas *et al*. and us predict that about 50% of transcripts in the retina have an altered structure compared to the reference (Supplementary Table S6). Moreover, the results of novel gene identification were generally consistent (27% of overlap), but with a relative increase of novel genes identified in our study (206 novel genes compared to 114 in Farkas *et al*.). We could not perform a detailed comparison with Li *et al*., as their data are not publicly available. They identified about 18K genes as expressed in human retina, which corresponds to about 80% of all the genes annotated in RefSeq. Our analysis was more stringent and identified about 13K genes.

Recently, single-cell transcriptome analysis of human retina has been performed to identify and categorise the different cell-types composing this complex tissue (45). One limitation of single-cell transcriptomics with current technologies is their limited resolution power in terms of the sample size and depth-of-coverage, resulting in a noisier signal for medium and low expressed transcripts (45). Hence, the bulk transcriptome analysis reported in the present manuscript led to the establishment of a reliable reference transcriptome that is very valuable to reconstruct co-regulation relationships among genes.

## CONCLUSIONS

We generated the most accurate and high-resolution atlas of gene expression and gene co-regulation in human retina to date, together with an online tool to quickly access and explore the data (http://retina.tigem.it). We believe that this atlas will represent a valuable resource for the research community at large and help in better elucidating pathophysiological processes in the human retina.

## DATA ACCESS

RNA sequencing aligned data (bam files) are made available through EBI's ArrayExpress (E-MTAB-4377). A custom website (http://retina.tigem.it) provides access to all of the results, including average coverage, inferred transcripts, genes’ and transcripts’ expression levels, and the gene co-expression network.

## Supplementary Material

SUPPLEMENTARY DATA
