# Supplementary material for: An atlas of gene expression and gene co-regulation in the human retina
Source: Nucleic Acids Res. 2016 May 27;44(12):5773–84. doi: 10.1093/nar/gkw486 (PMC4937338; doi:10.1093/nar/gkw486)
Supplement: SUPPLEMENTARY DATA [file supp_44_12_5773__index.html]

An atlas of gene expression and gene co-regulation in the human retina — An atlas of gene expression and gene co-regulation in the human retina — SUPPLEMENTARY DATA 

# An atlas of gene expression and gene co-regulation in the human retina

## SUPPLEMENTARY DATA

- SUPPLEMENTARY DATA
- SUPPLEMENTARY DATA
- SUPPLEMENTARY DATA
- SUPPLEMENTARY DATA
- SUPPLEMENTARY DATA
- SUPPLEMENTARY DATA
- SUPPLEMENTARY DATA
- SUPPLEMENTARY DATA
- SUPPLEMENTARY DATA
- SUPPLEMENTARY DATA
- SUPPLEMENTARY DATA
- SUPPLEMENTARY DATA
- SUPPLEMENTARY DATA
- SUPPLEMENTARY DATA
