# Supplementary material for: An atlas of gene expression and gene co-regulation in the human retina
Source: Nucleic Acids Res. 2016 May 27;44(12):5773–84. doi: 10.1093/nar/gkw486 (PMC4937338; doi:10.1093/nar/gkw486)

Supplementary Figure S1: Pre- and post-intrasample normalization

Panels A and B show the average expression (y-axis) of genes of a given length (x-axis) respectively before and after full-quantile normalization (see methods). Panel C shows the average expression (y-axis) of genes of a given GC-content percentage (x-axis). Lines represent sample lowess summarization.

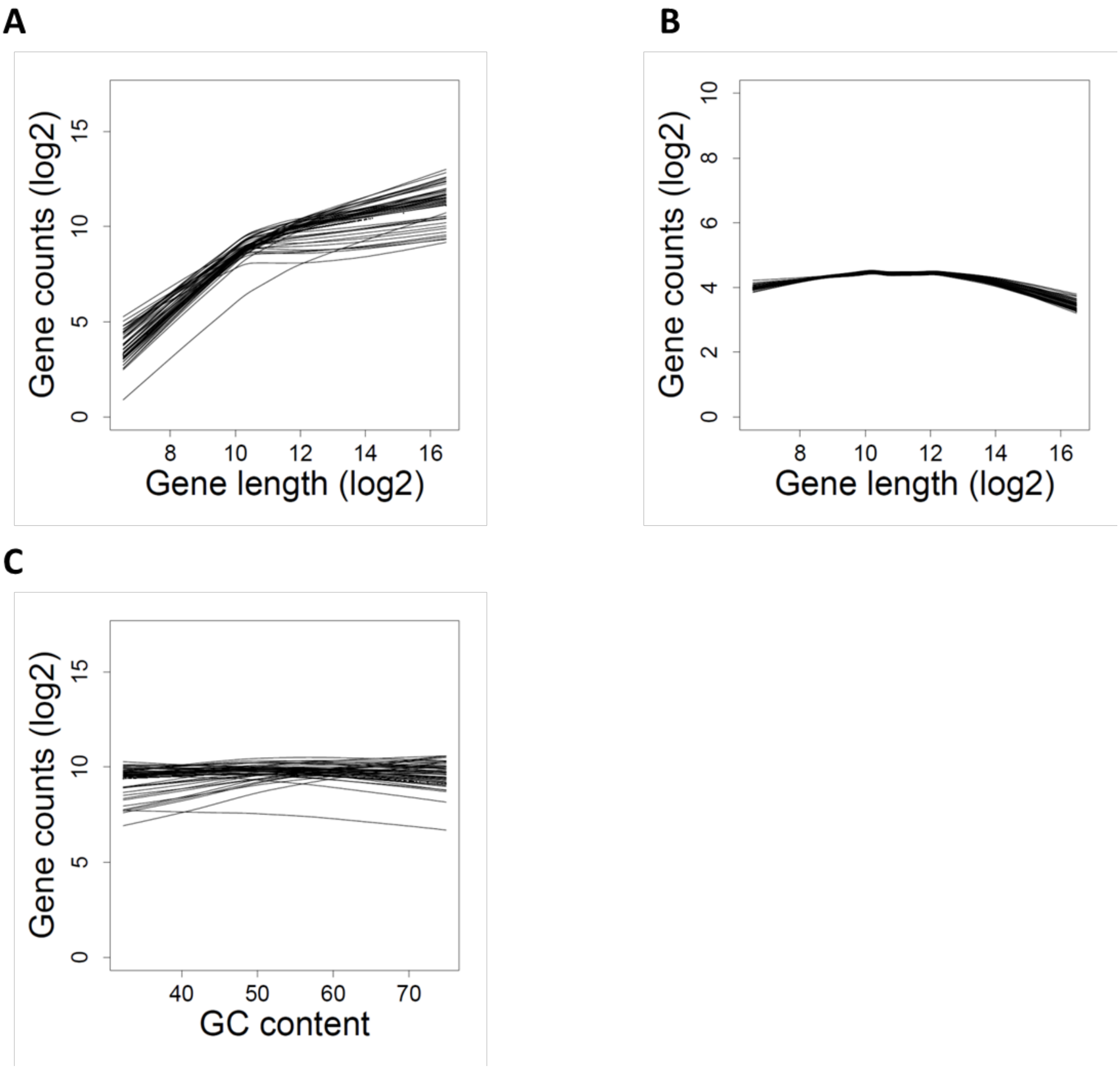

Supplementary Figure S2: Pre- and post-intersample TMM

Gene expression distribution (y-axis) per sample (x-axis) before (panel A) and after (panel B) Trimmed Mean of M values normalization (TMM)

A: Before TMM

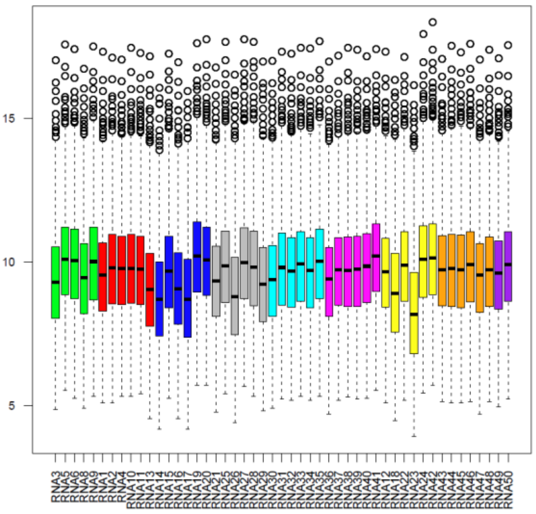

B: After TMM

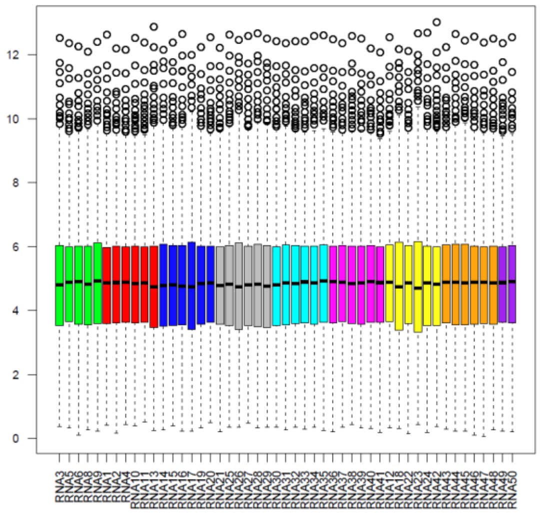

Supplement: SUPPLEMENTARY DATA [file supp_gkw486_nar-00602-z-2016-File007.pdf]
