# Supplementary material for: An atlas of gene expression and gene co-regulation in the human retina
Source: Nucleic Acids Res. 2016 May 27;44(12):5773–84. doi: 10.1093/nar/gkw486 (PMC4937338; doi:10.1093/nar/gkw486)

Supplementary Figure S3: (A) Precision-recall and (B) Receiver-Operator Characteristic (ROC) curve of the Gene network with the STRING interactions

Agreement between gene co-expression network and protein-protein interactions as derived from STRING database.

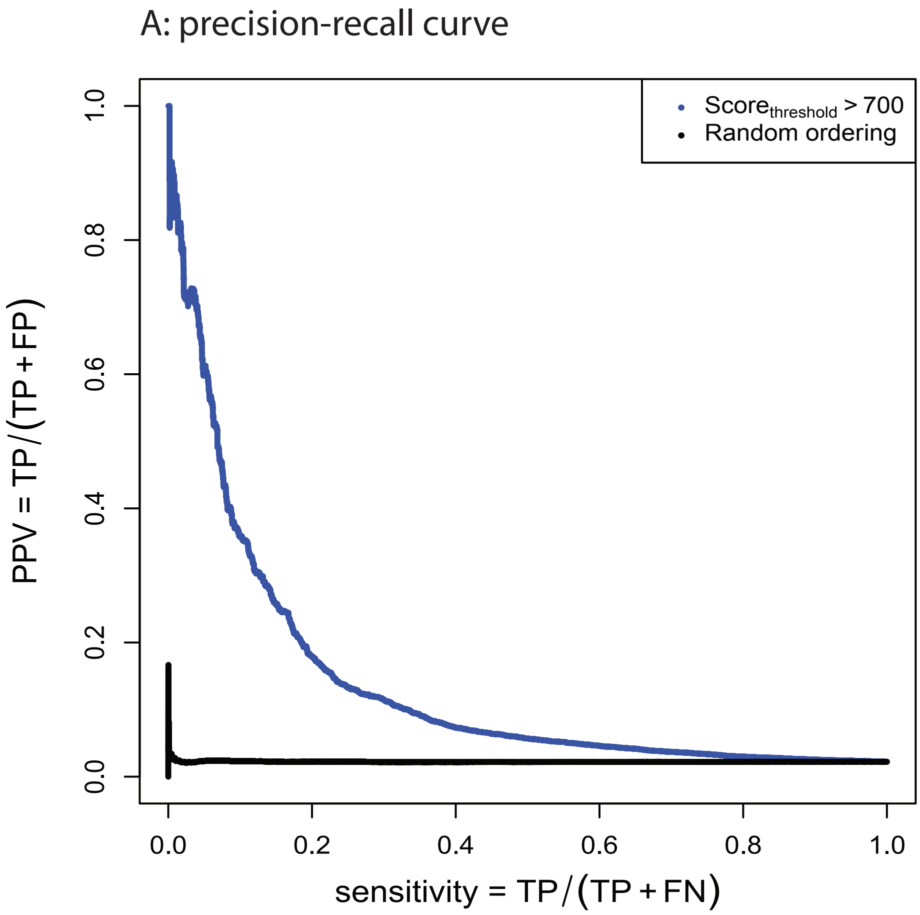

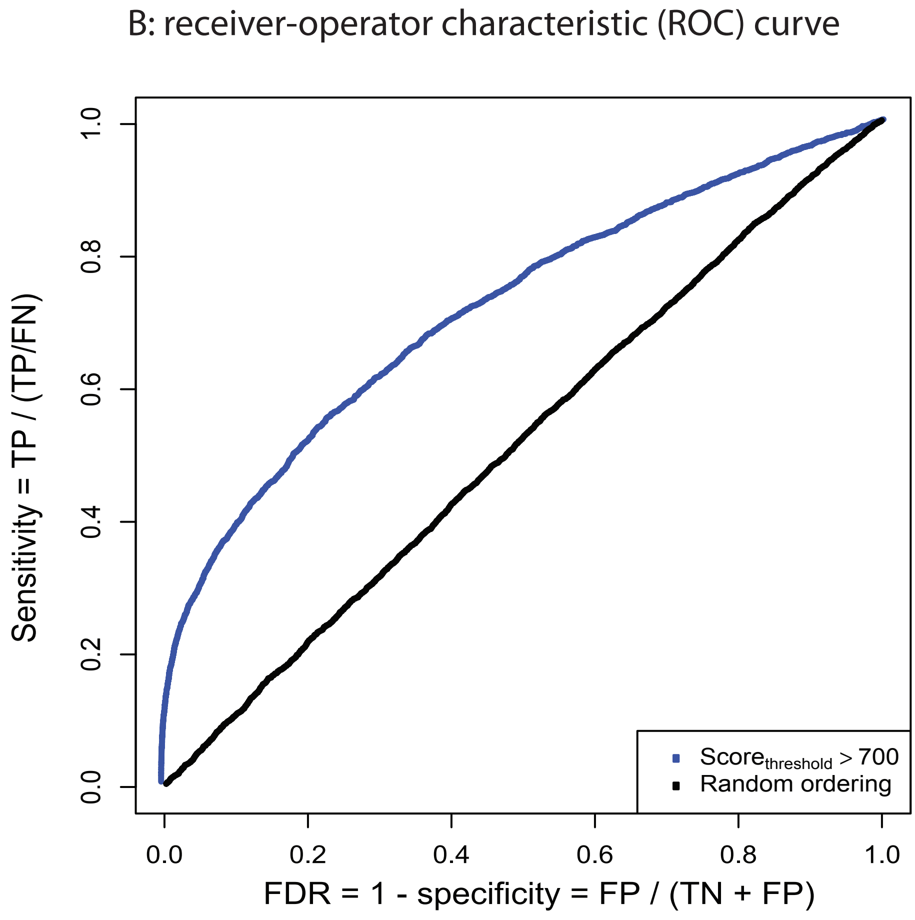

Supplement: SUPPLEMENTARY DATA [file supp_gkw486_nar-00602-z-2016-File008.pdf]
