# Supplementary material for: An atlas of gene expression and gene co-regulation in the human retina
Source: Nucleic Acids Res. 2016 May 27;44(12):5773–84. doi: 10.1093/nar/gkw486 (PMC4937338; doi:10.1093/nar/gkw486)

Supplementary Figure S4: Total and mapped fragments

A: Number of fragments (reads) generated per sample and mapped on reference genome (hg19)

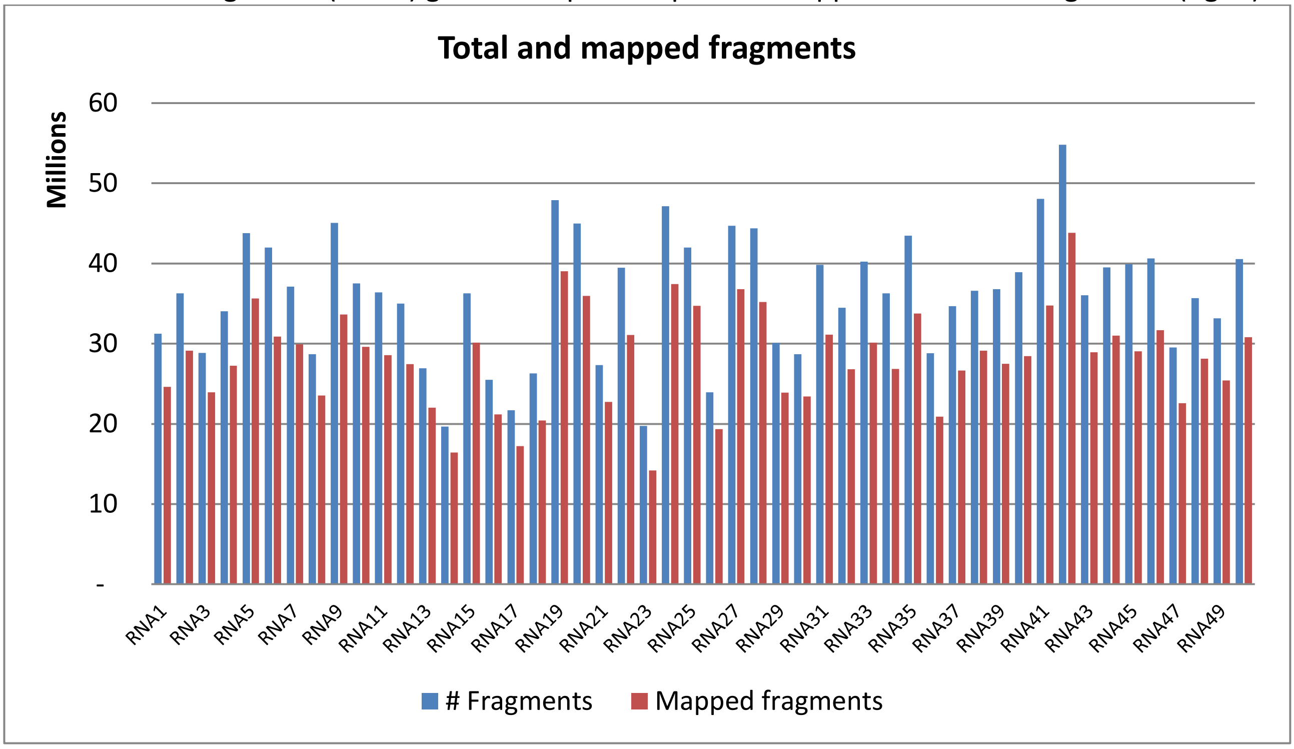

B: Sum of fragment length mapped on reference genome (hg19)

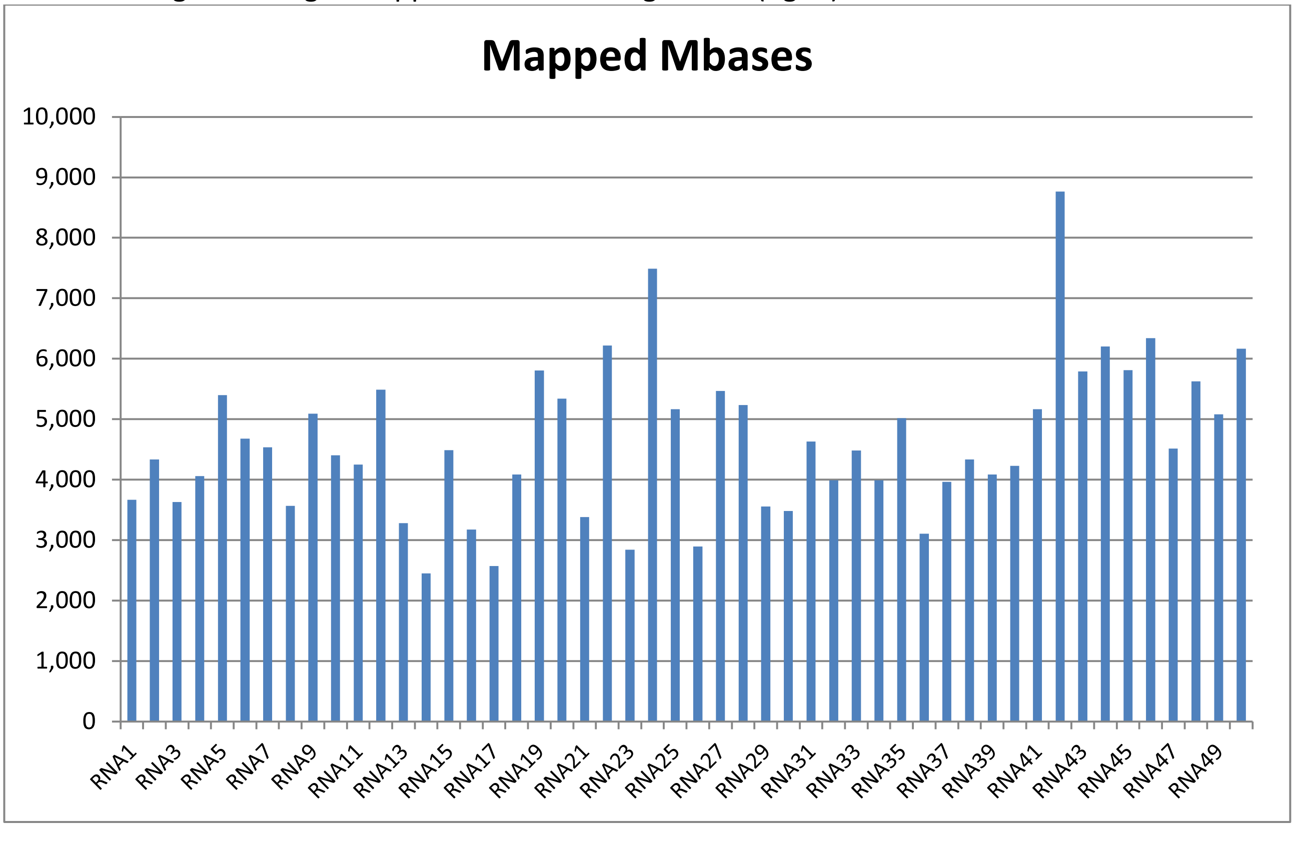

Supplementary Figure S5: Fraction of the genome that is transcribed in retina

Total length of the genome that we found expressed. Left bar represents regions with enough coverage and right bar regions covered by transcripts. The blue part is genome already known to be transcribed (overlap with Gencode transcripts) and the red part new transcript.

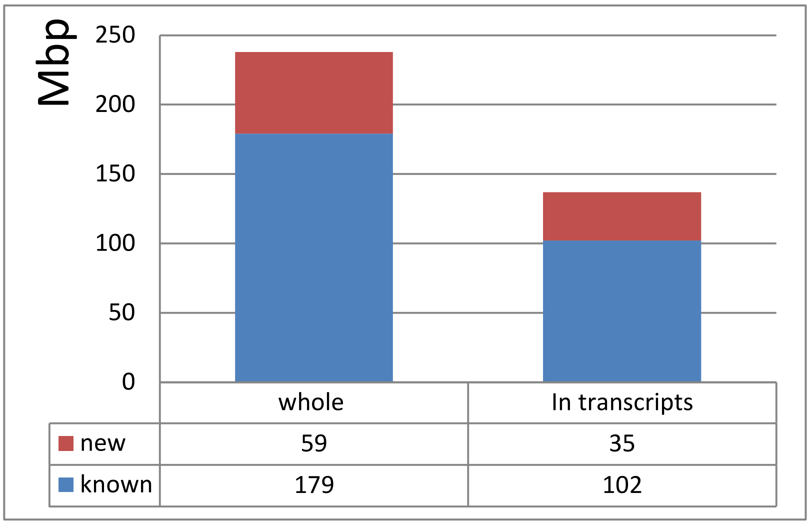

Supplementary Figure S6: example of inferred transcript structures on non-standard chromosomes

Alignment of two genes on non-standard genome contigs. In black are marked the inferred transcripts and in violet the known transcripts.

A: chrUn_gl000219:48941-106062

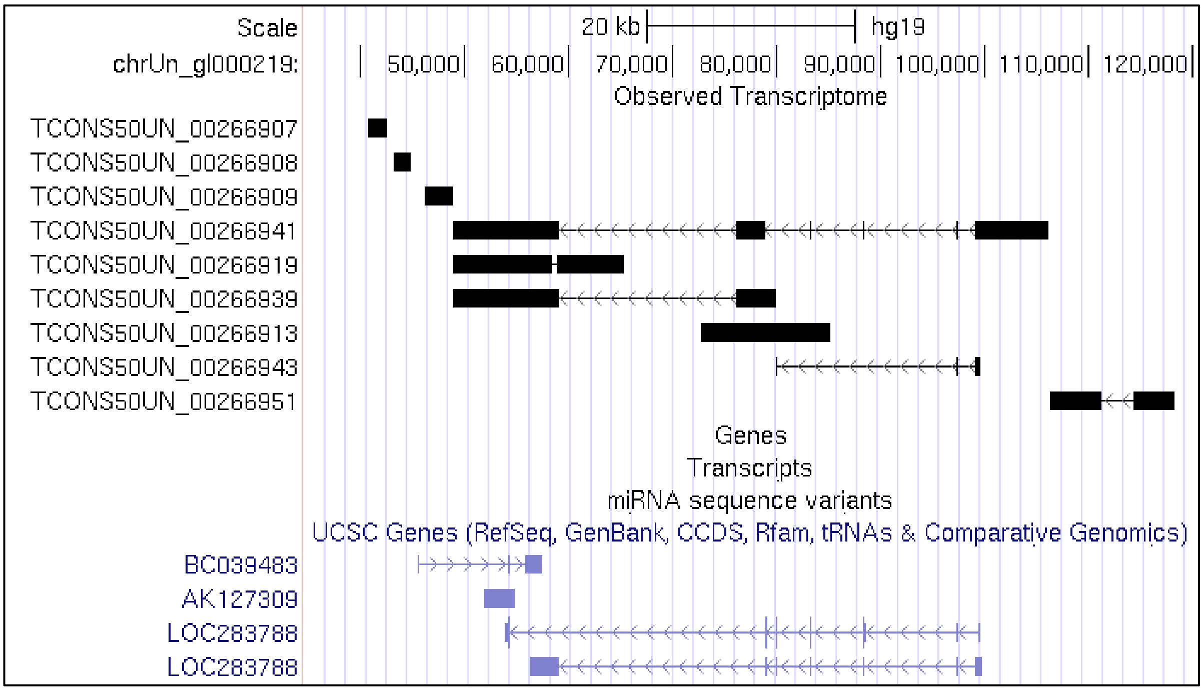

B: chr4_gl000194_random:55644-112812

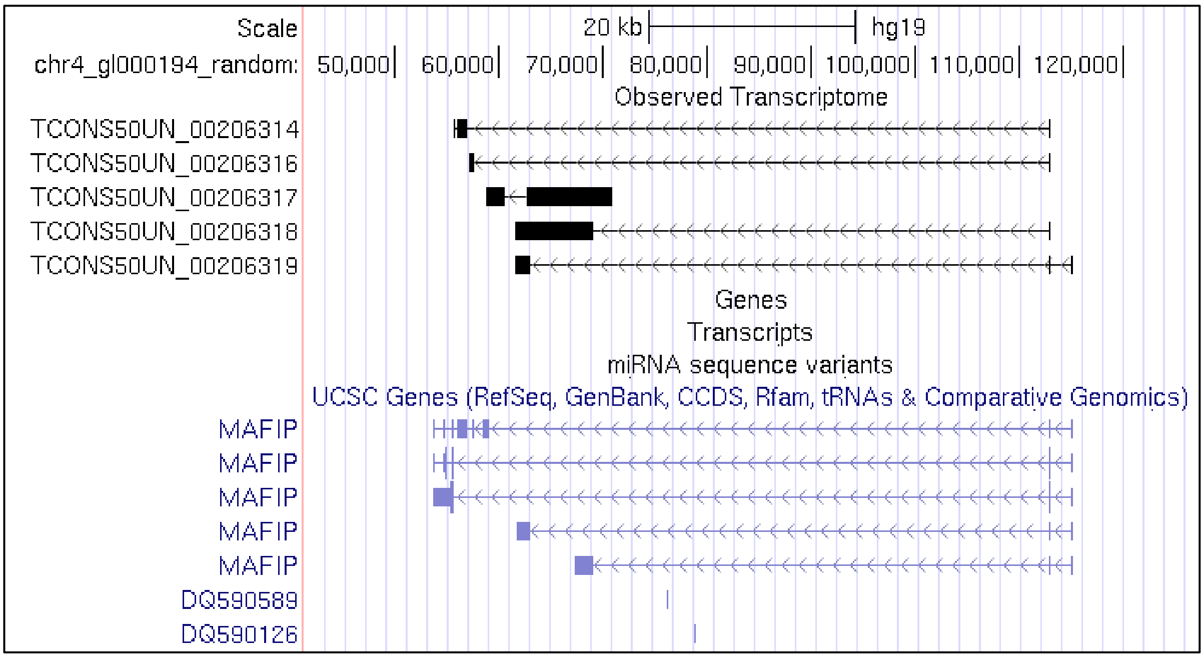

Supplementary Figure S7: Percent of transcripts with alterations stratified by relative and absolute expression

Percentage of transcripts that perfectly matched Gencode. Plot A stratifies transcripts by their absolute expression in 10 bins. Plot B stratifies the transcripts for their perceptual contribution within each gene. The higher the absolute and relative expression the lower is the occurrence of alteration compared to Gencode.

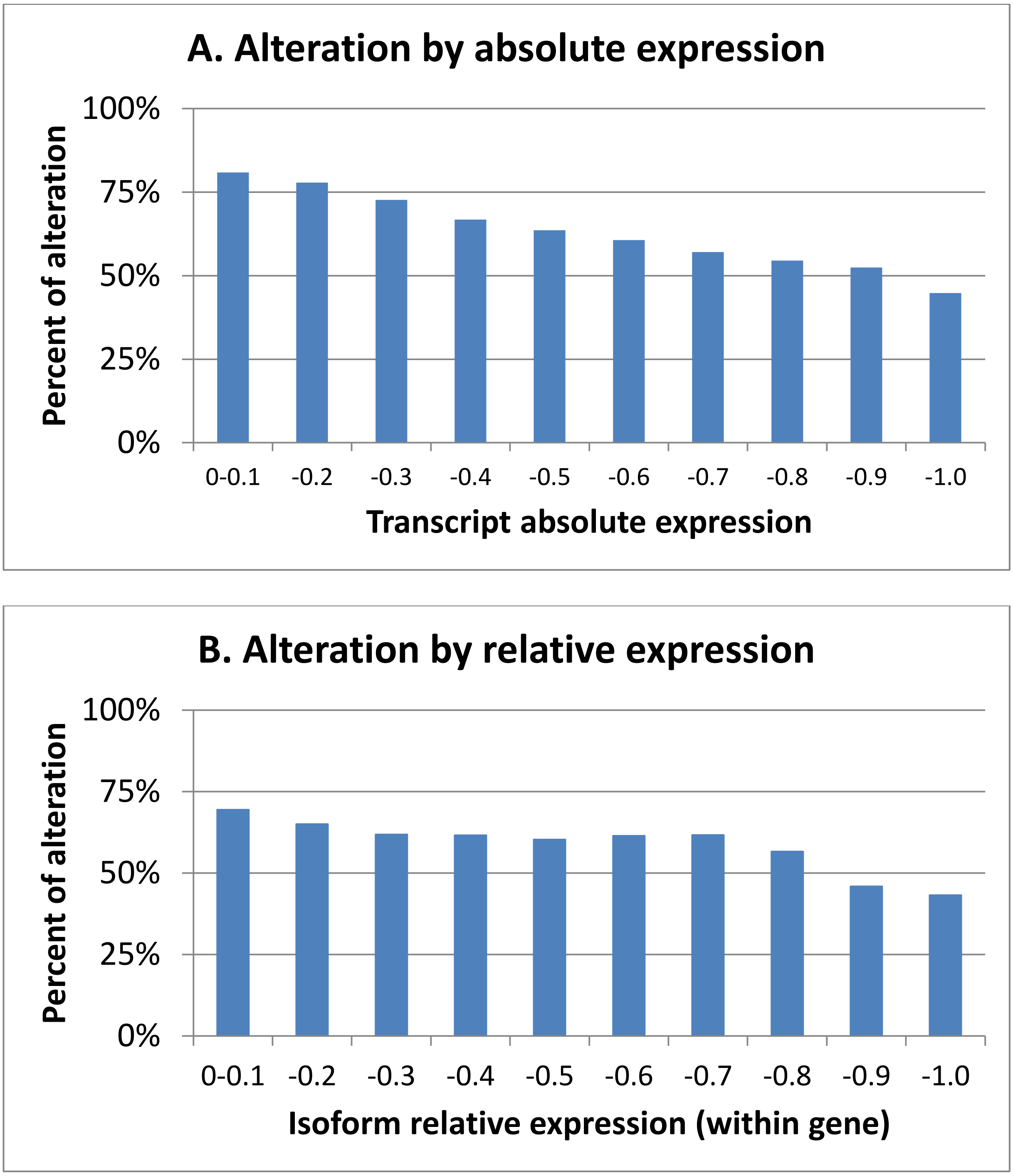

Supplementary Figure S8

Gene expression of the ObsT stratified for to their comparison with Gencode. “New genes” are those that were identified in this study for the first time. “New isoforms” are genes that have no isoforms matching a Gencode transcript. “Known isoforms” are genes that have isoforms matching a Gencode transcript.

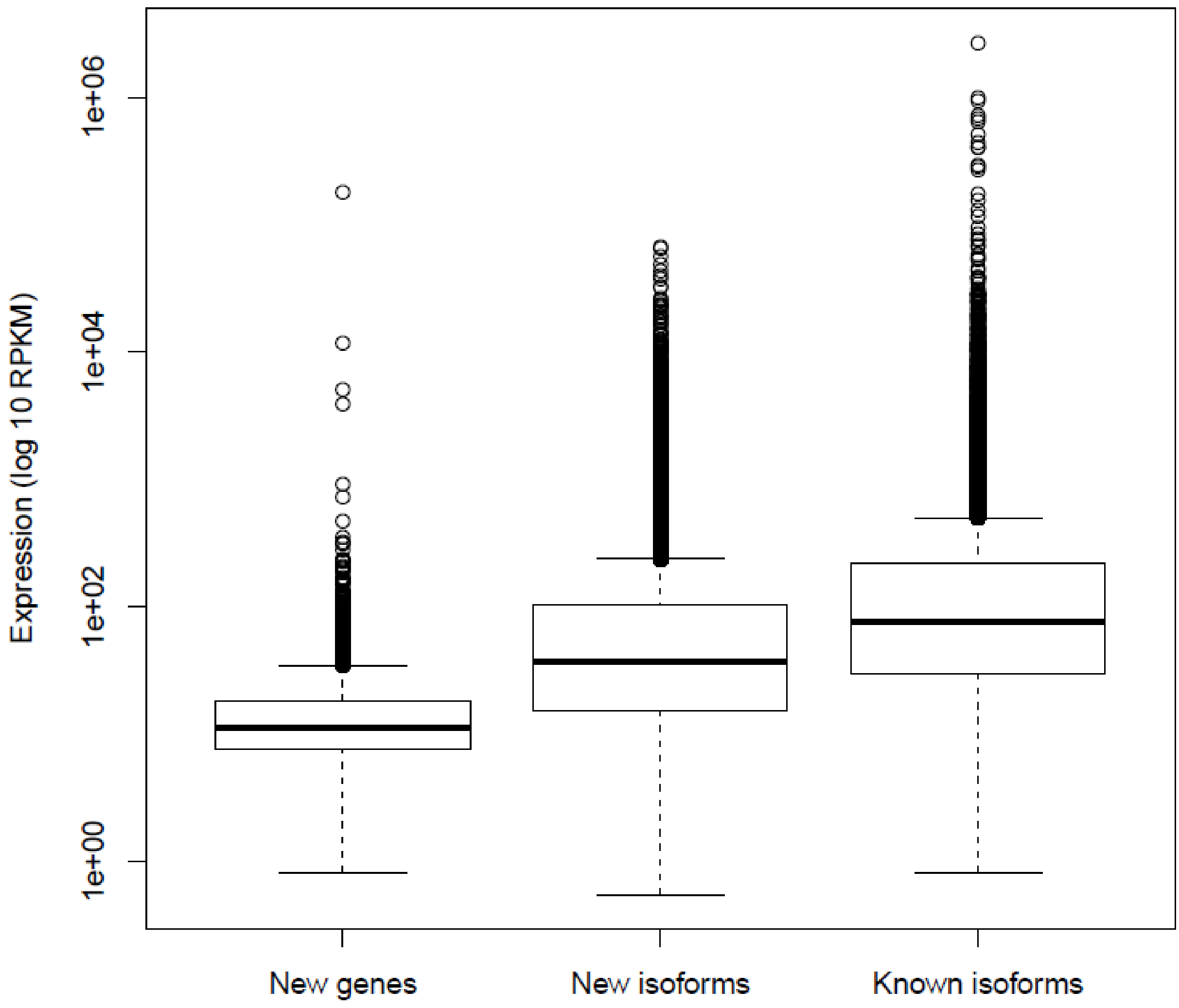

Supplementary Figure S9: Effect of confounding factors on the first two principal components of the gene expression

A: Multidimensional Scaling Plot (MDS) generated by edgeR using top 500 genes and colored according to sequencing lanes. There is no apparent batch effect.

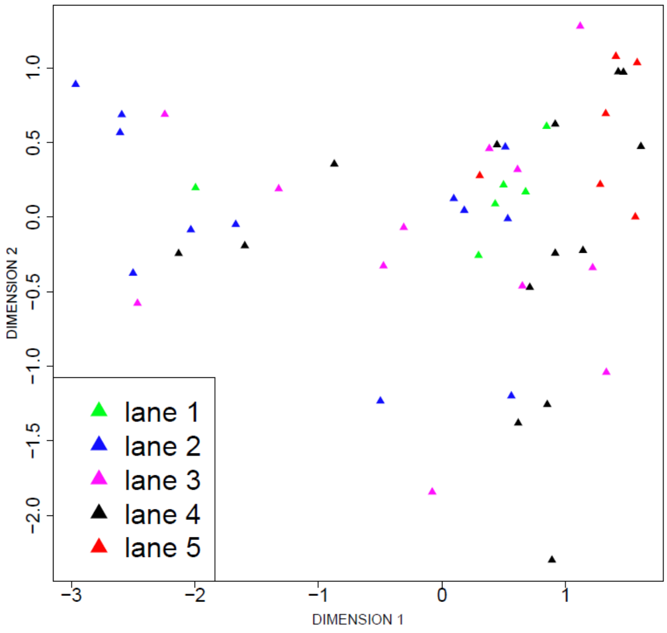

B: Multidimensional Scaling Plot (MDS) generated by edgeR using X- and Y-chromosome genes and colored according to sequencing lanes. A clear gender clustering is apparent.

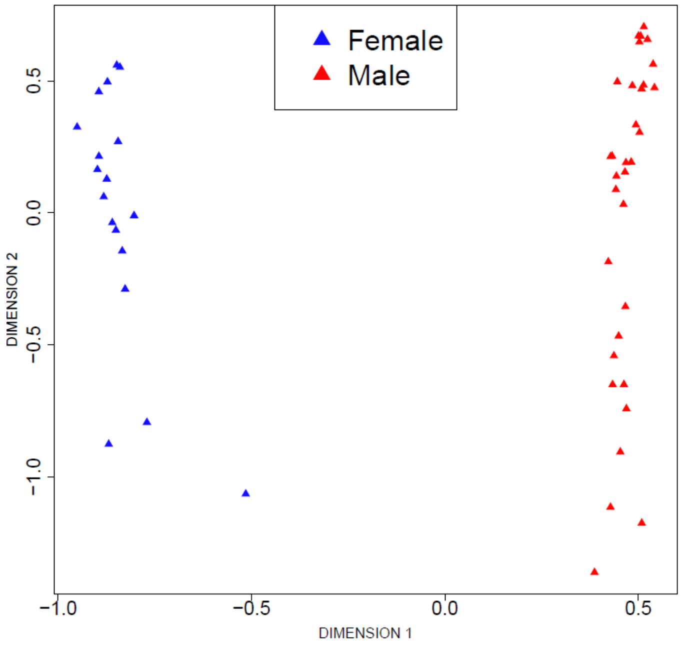

Supplement: SUPPLEMENTARY DATA [file supp_gkw486_nar-00602-z-2016-File009.pdf]
