# Supplementary material for: An atlas of gene expression and gene co-regulation in the human retina
Source: Nucleic Acids Res. 2016 May 27;44(12):5773–84. doi: 10.1093/nar/gkw486 (PMC4937338; doi:10.1093/nar/gkw486)
Supplement: SUPPLEMENTARY DATA [file supp_gkw486_nar-00602-z-2016-File010.pdf]

Supplementary Figure S10: gene expression of selected genes that are mostly expressed in cellular subpopulations.

Panels show the overall gene expression (gray) versus the expression of single gene (blue or orange). X-axis reports level of gene expression, y-axis reports density. Blue is for Photoreceptor genes, orange for RPE genes. Usually, photoreceptor genes are more expressed and RPE gene are less expressed than the rest of genes.

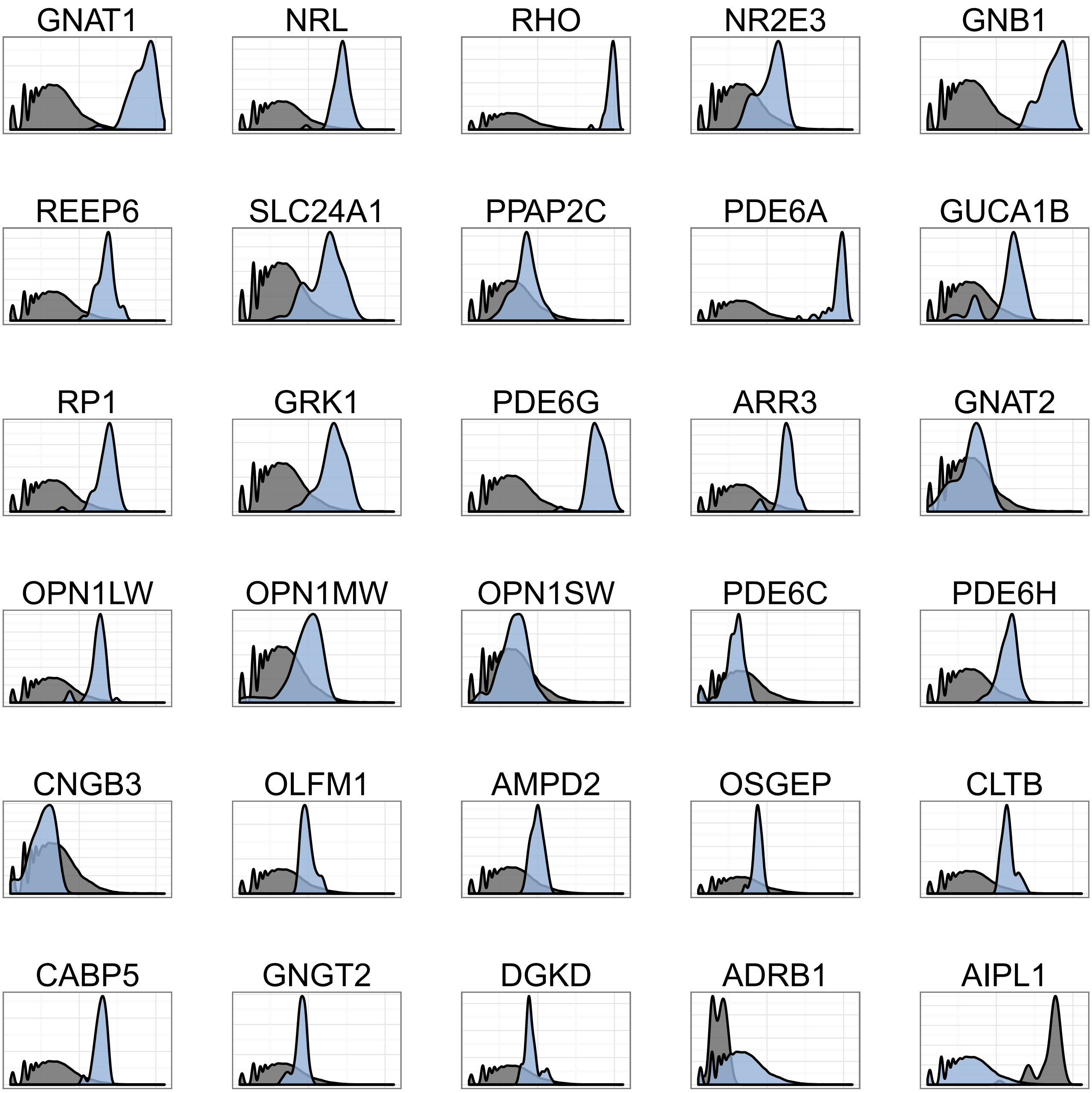

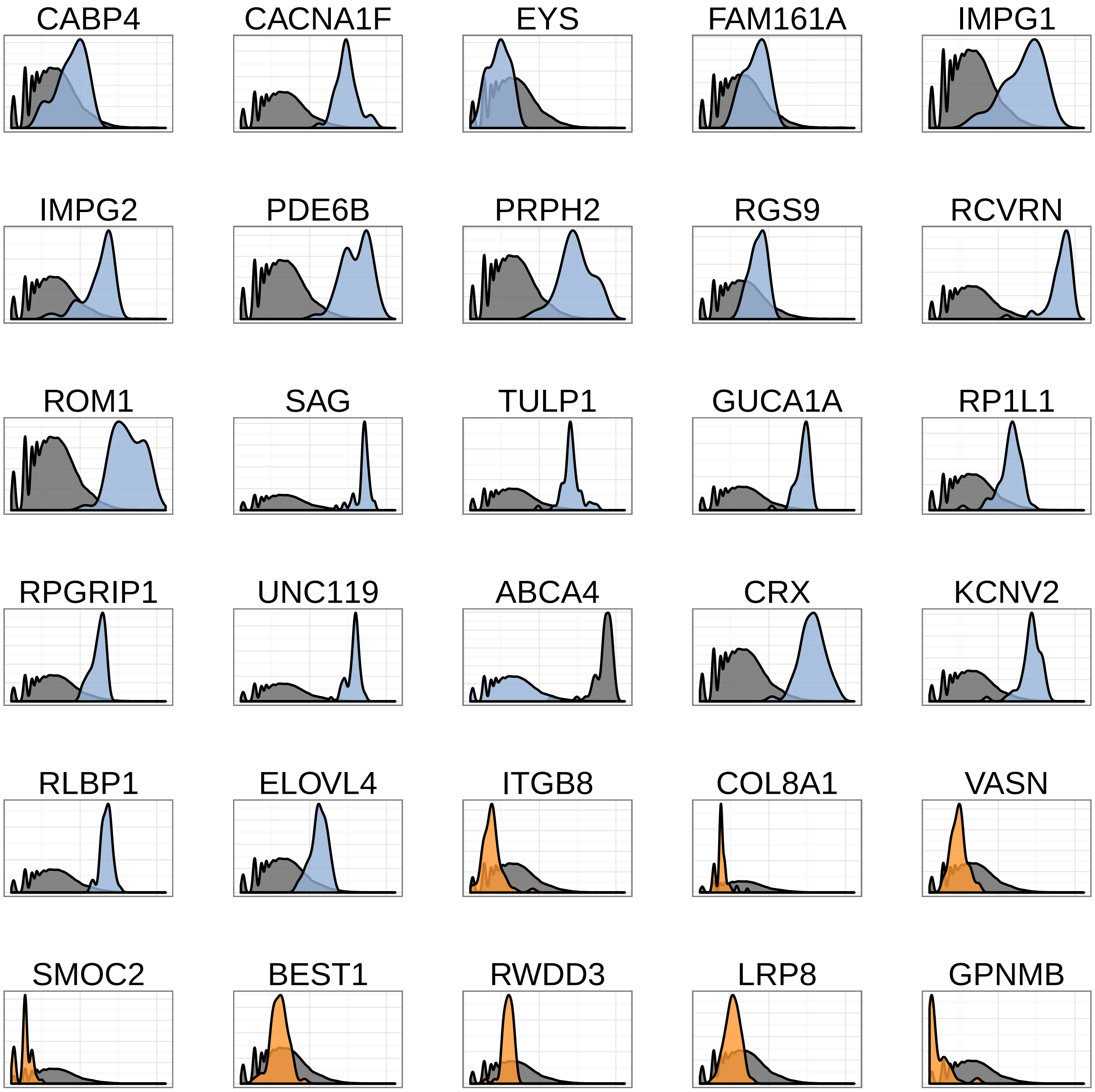
CABP4
CACNA1F
EYS
FAM161A
IMPG1
IMPG2
PDE6B
PRPH2
RGS9
RCVRN
ROM1
SAG
TULP1
GUCA1A
RP1L1
RPGRIP1
UNC119
ABCA4
CRX
KCNV2
RLBP1
ELOVL4
ITGB8
COL8A1
VASN
SMOC2
BEST1
RWDD3
LRP8
GPNMB
