# Supplementary material for: An atlas of gene expression and gene co-regulation in the human retina
Source: Nucleic Acids Res. 2016 May 27;44(12):5773–84. doi: 10.1093/nar/gkw486 (PMC4937338; doi:10.1093/nar/gkw486)

Supplementary Figure S11: distribution of expressed genes by chromosome.

Overall gene expression stratified by chromosome. Genes mapping to mitochondrial chromosome contributed largely to overall retina gene expression.

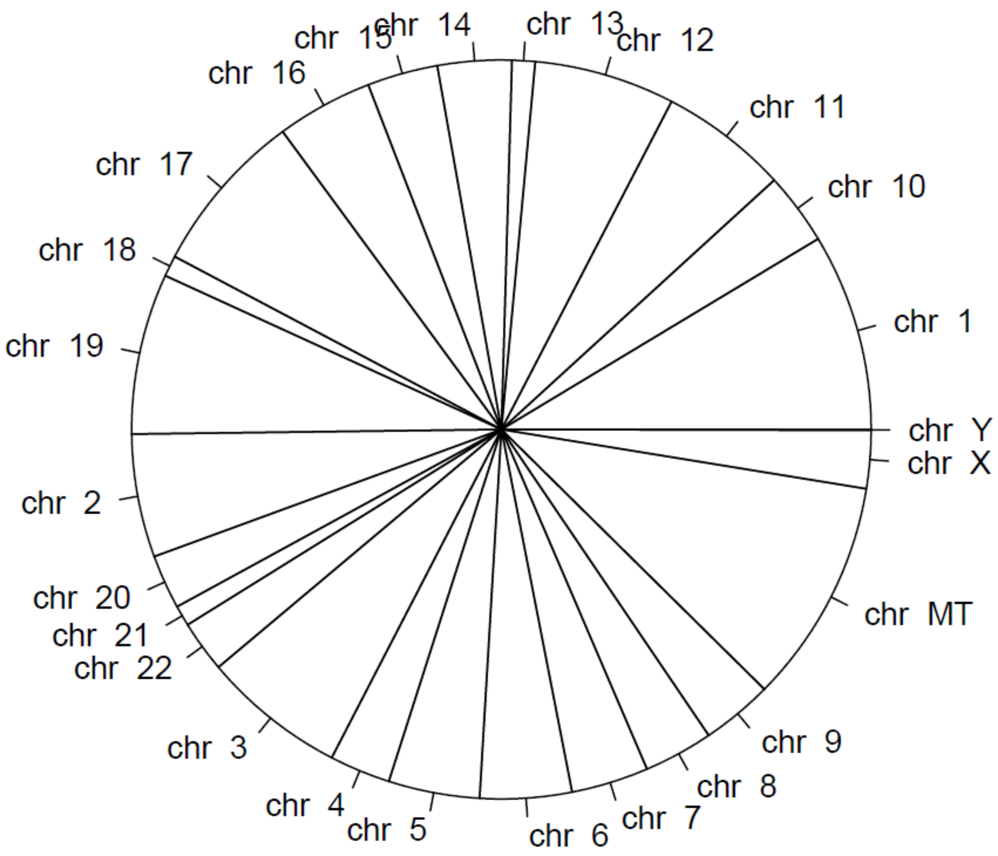

Supplementary Figure S12: GSEA top enriched GO categories for the most-expressed genes

Gene Set Enrichment Analysis of GO-categories (MSIG C5) against gene expression.

A: Detection of External Stimulus

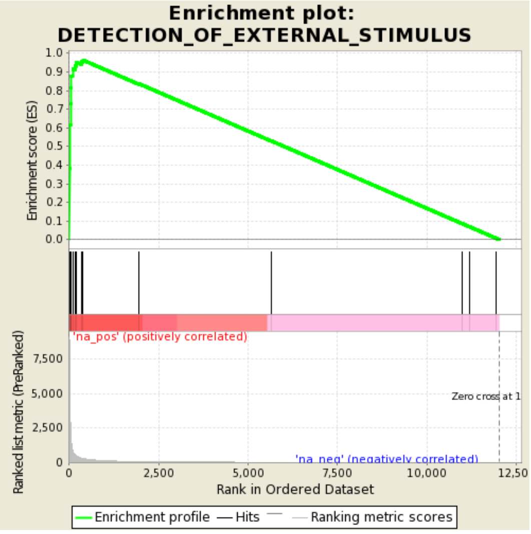

B: Response to light stimulus

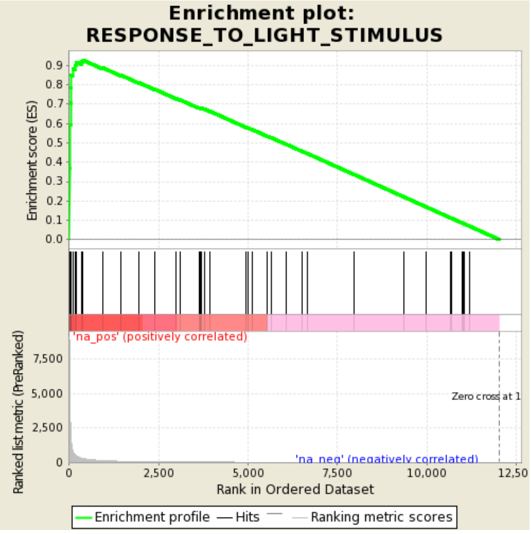

C: Sensory perception

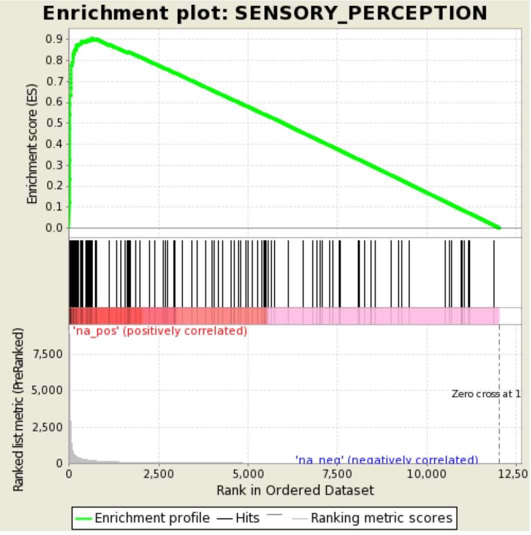

Gene-set	SIZE	ES	NES	NOM p-val	FDR q-val	FWER p-val
DETECTION_OF_STIMULUS	46	0.97	1.49	0	0	0
DETECTION_OF_EXTERNAL_STIMULUS	22	0.98	1.47	0	0.00349	0.007
DETECTION_OF_STIMULUS_INVOLVED_IN_SENSORY_PERCEPTION	20	0.97	1.46	0	0.010968	0.032
DETECTION_OF_ABIOTIC_STIMULUS	18	0.97	1.45	0	0.01371	0.051
RESPONSE_TO_LIGHT_STIMULUS	45	0.93	1.43	0	0.096122	0.381
SENSORY_PERCEPTION	188	0.91	1.43	0	0.080102	0.381
RESPONSE_TO_RADIATION	58	0.91	1.42	0	0.085039	0.453
RHODOPSIN_LIKE_RECEPTOR_ACTIVITY	133	0.88	1.39	0	0.23993	0.866
CARBOHYDRATE_KINASE_ACTIVITY	15	0.94	1.39	0.012	0.226234	0.881
STRUCTURAL_CONSTITUENT_OF_RIBOSOME	80	0.88	1.38	0	0.327751	0.963

Supplementary Figure S13: Expression of retina-important genes

Gene Set Enrichment Analysis of Photoreceptor and RetNet genes against gene expression.

A: Photoreceptors

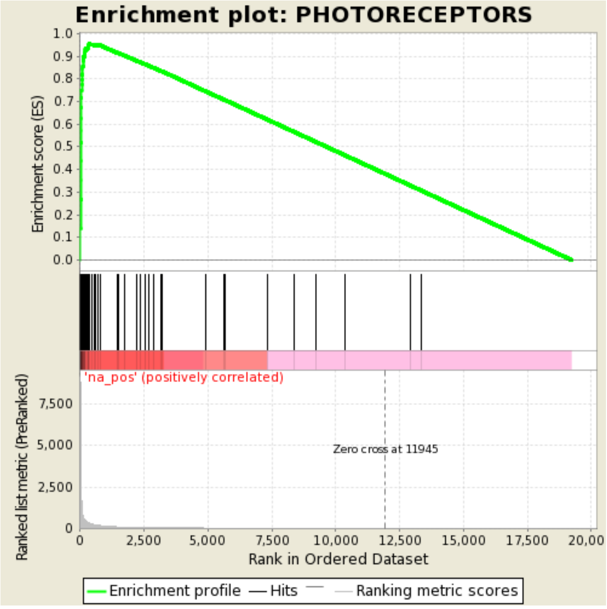

B: RetNet

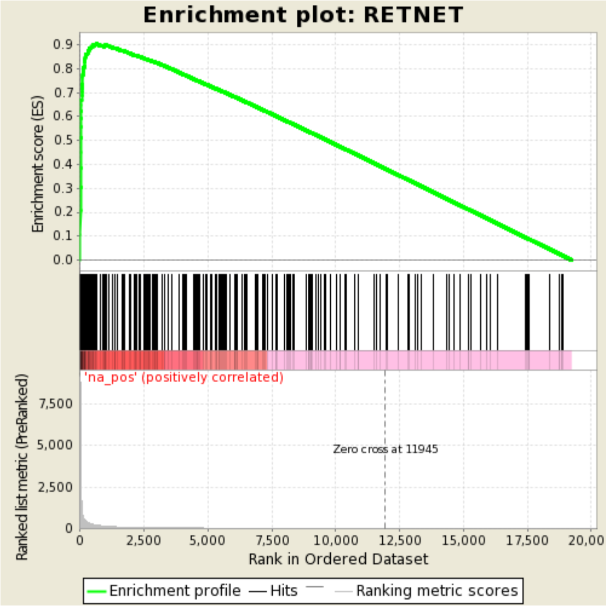

Supplementary Figure S14: GSEA top enriched GO categories for the most and less variably expressed genes

Gene Set Enrichment Analysis of GO-categories (MSIG C5) against gene expression variability (CV).

A. Most variable

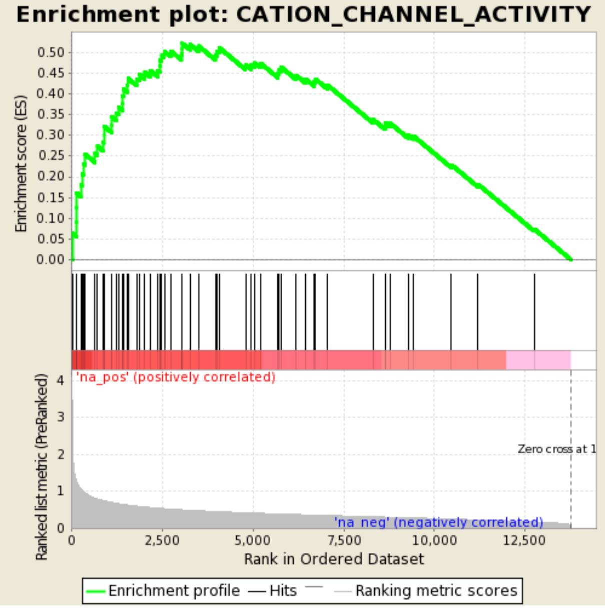

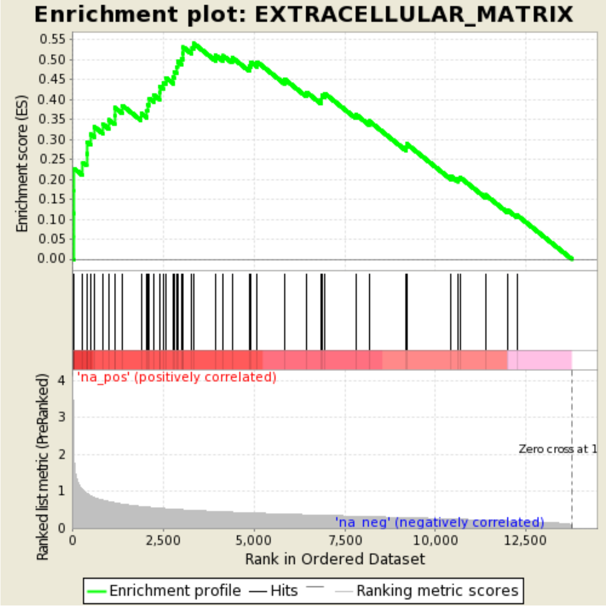

B. **Less variable**

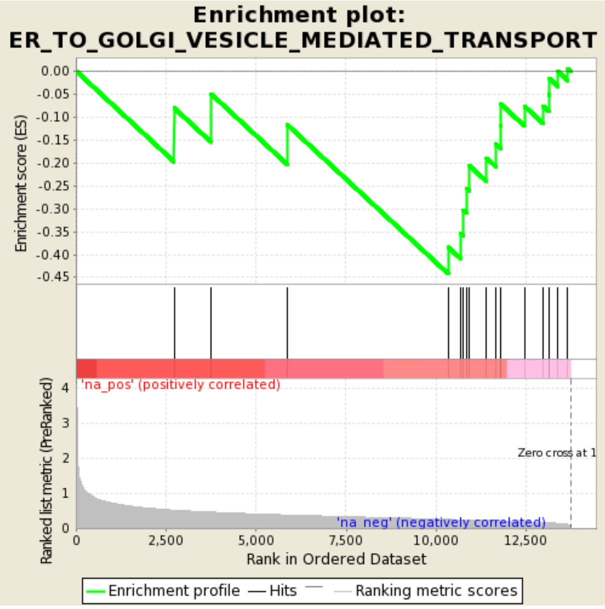

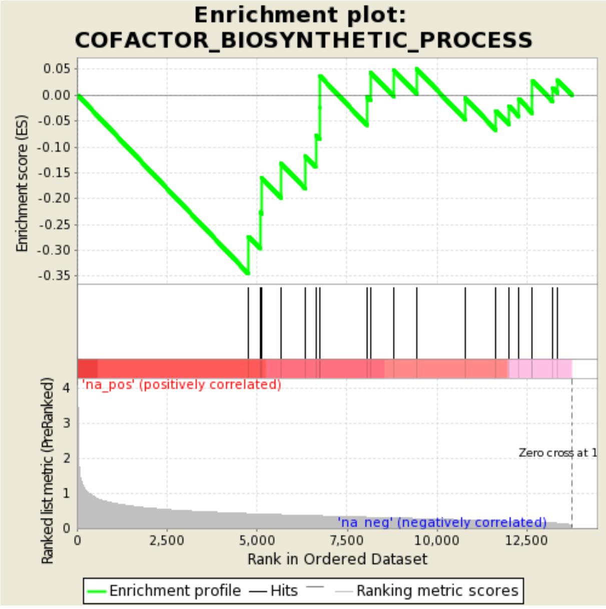

Supplementary Figure S15: Overlap between ObsT and RefT

A: overlap between the genes that have been found as expressed in the ObsT and the RefT. Areas are not proportional to the number of genes.

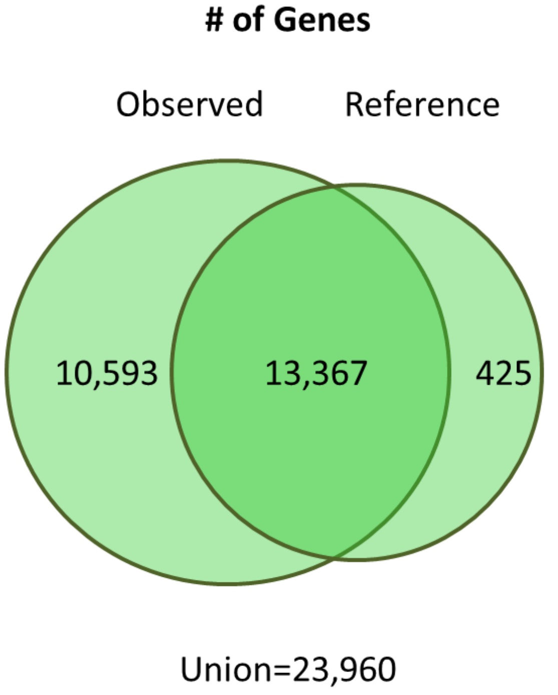

B: Scatterplot of the estimated gene expression in the RefT (y-axis) and ObsT (x-axis).

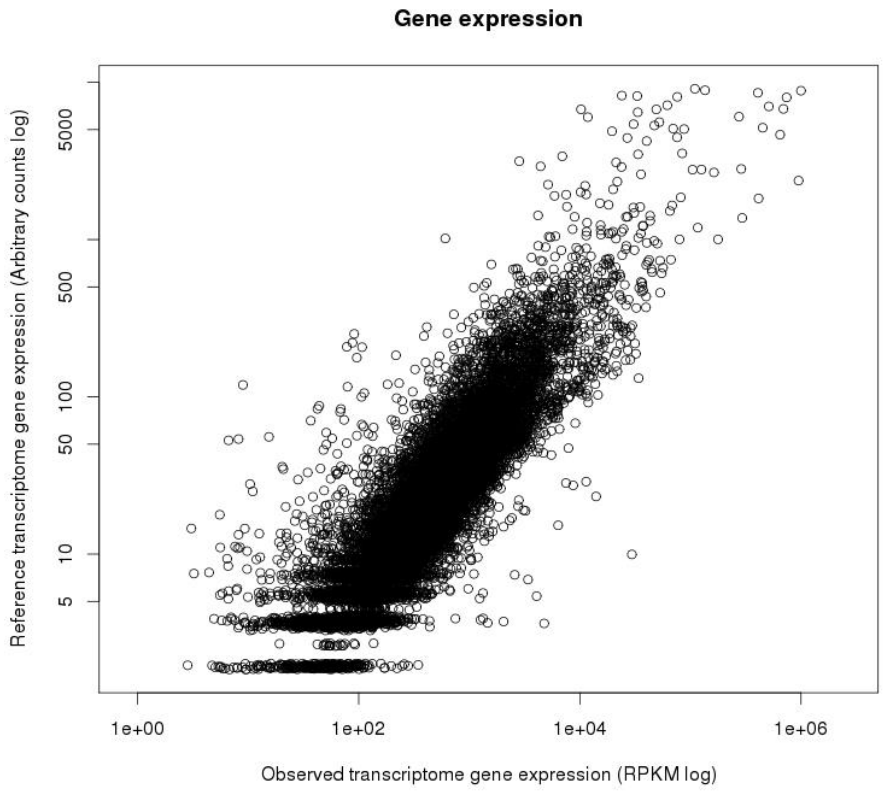

Supplementary Figure S16: Expression level of ObsT genes stratified by their presence also in the RefT.

The “Not in Reference Transcriptome” box shows the expression of ObsT-only genes. The “In Reference Transcriptome” shows the expression of ObsT and RefT genes.

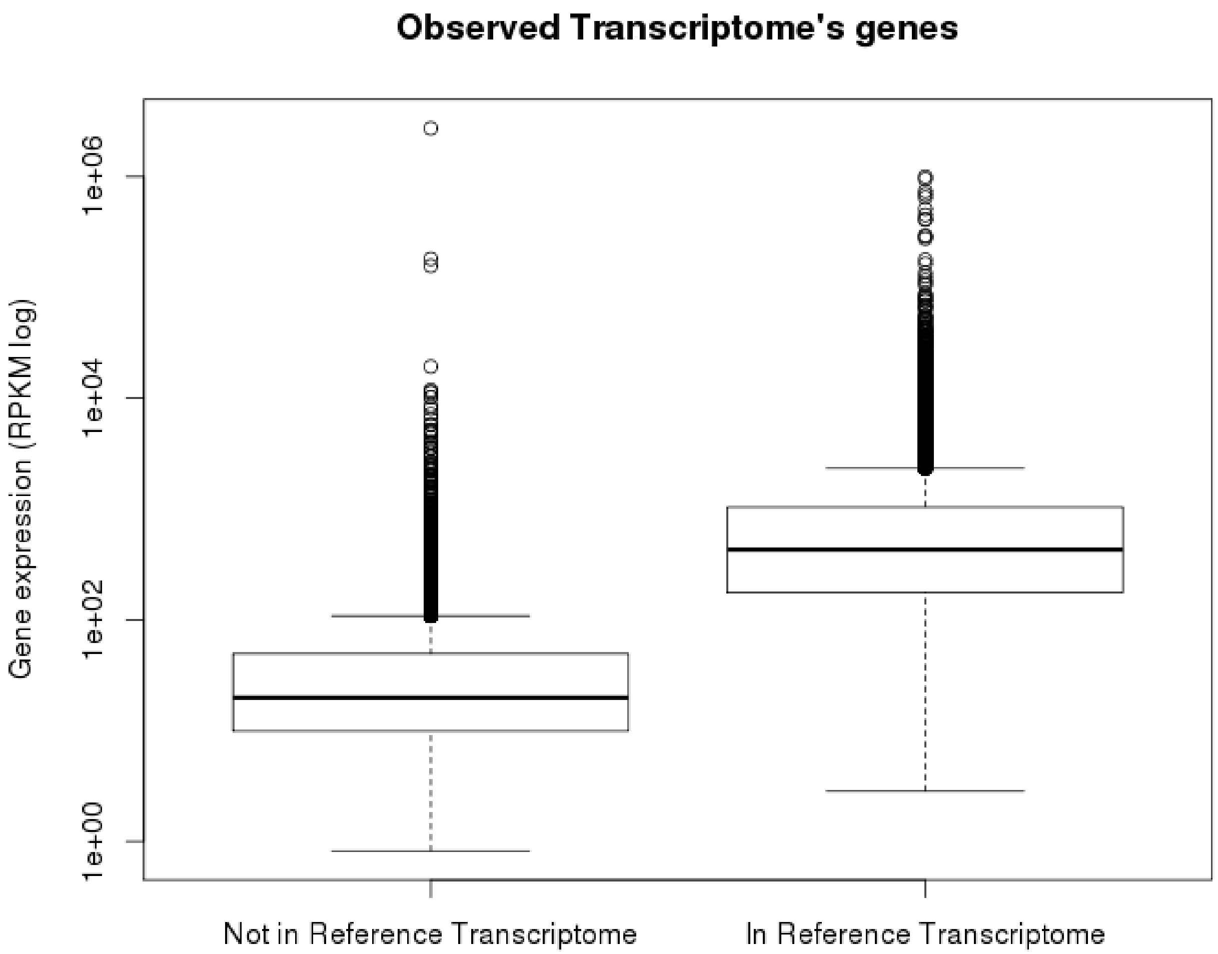

Supplementary Figure S17: Alignment of RNAseq data on two regions of mitochondrial genome

UCSC genome browser snapshots of two mitochondrial chromosome regions. Three tracks are shown: on top the coverage, on the middle the aligned reads, and on bottom the known genes. The plots show that the genic regions are more covered than intergenic regions, consistent with a proper enrichment of transcripts rather than genomic DNA. Panel A shows region chrM:1-8000 and B chrM:4600-6600.

A

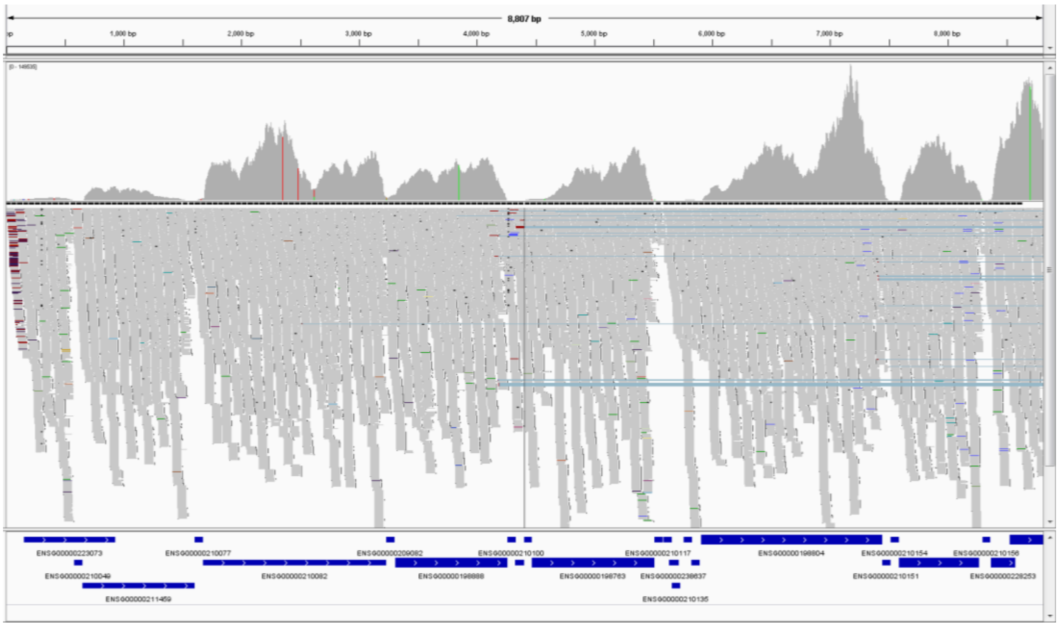

B

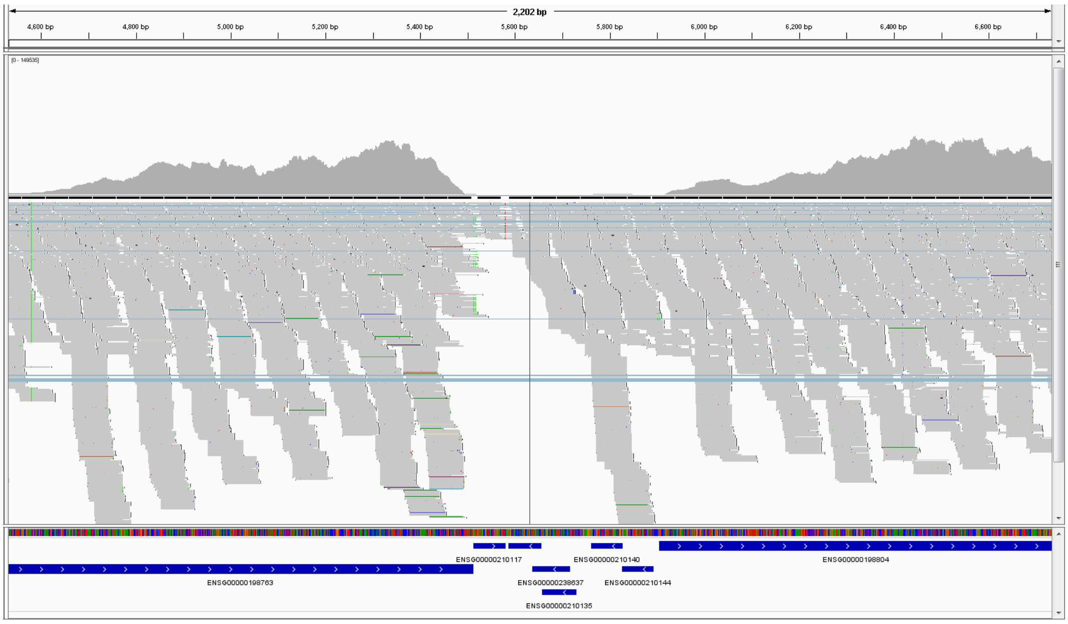

Supplement: SUPPLEMENTARY DATA [file supp_gkw486_nar-00602-z-2016-File011.pdf]
