# Supplementary material for: An atlas of gene expression and gene co-regulation in the human retina
Source: Nucleic Acids Res. 2016 May 27;44(12):5773–84. doi: 10.1093/nar/gkw486 (PMC4937338; doi:10.1093/nar/gkw486)
Supplement: SUPPLEMENTARY DATA [file supp_gkw486_nar-00602-z-2016-File012.docx]

# Supplementary Table S1: Donor description and sample features

| Sample | Gender | Age | Cause of death | Total post-mortem time (T) |
| --- | --- | --- | --- | --- |
| 1 | Female | 68 | Neoplastic Disease | 25h 15min |
| 2 | Male | 52 | Neoplastic Disease | 21h 30min |
| 3 | Male | 67 | Neoplastic Disease | 22h 50min |
| 4 | Female | 70 | Neoplastic Disease | 24h 55min |
| 5 | Male | 72 | Cardiovascular Disease | 25h 06min |
| 6 | Male | 60 | Neoplastic Disease | 25h 25min |
| 7 | Male | 50 | Cardiovascular Disease | 26h |
| 8 | Male | 71 | Neoplastic Disease | 21h 35min |
| 9 | Female | 71 | Neoplastic Disease | 14h 15min |
| 10 | Male | 70 | Neoplastic Disease | 23h 25min |
| 11 | Male | 49 | Injury/Poisoning | 23h 30min |
| 12 | Female | 63 | Neoplastic Disease | 19h 15min |
| 13 | Female | 59 | Neoplastic Disease | 24h 40min |
| 14 | Female | 69 | Neoplastic Disease | 21h |
| 15 | Male | 71 | Neoplastic Disease | 22h 15min |
| 16 | Male | 64 | Neoplastic Disease | 26h |
| 17 | Male | 42 | Injury/Poisoning | 21h 50min |
| 18 | Male | 68 | Neoplastic Disease | 21h 42min |
| 19 | Female | 50 | Neoplastic Disease | 19h 35min |
| 20 | Male | 64 | Digestive disease | 22h |
| 21 | Male | 71 | Neoplastic Disease | 25h |
| 22 | Female | 71 | Neoplastic Disease | 24h |
| 23 | Male | 63 | Neoplastic Disease | 13h |
| 24 | Male | 64 | Neoplastic Disease | 21h 30min |
| 25 | Male | 46 | Neoplastic Disease | 23h 45min |
| 26 | Female | 63 | Neoplastic Disease | 18h |
| 27 | Female | 49 | Neoplastic Disease | 7h 15min |
| 28 | Female | 69 | Cardiovascular Disease | 21h 50min |
| 29 | Male | 48 | Neoplastic Disease | 25h 30min |
| 30 | Male | 62 | Digestive disease | 25h 30min |
| 31 | Female | 71 | Neoplastic Disease | 20h |
| 32 | Female | 54 | Neoplastic Disease | 18h 29min |
| 33 | Male | 65 | Cardiovascular Disease | 21h |
| 34 | Male | 59 | Neoplastic Disease | 22h 45min |
| 35 | Male | 59 | Neoplastic Disease | 15h 10min |
| 36 | Male | 61 | Cardiovascular Disease | 19h 50min |
| 37 | Male | 59 | Cardiovascular Disease | 22h 45min |
| 38 | Male | 65 | Neoplastic Disease | 23h 15min |
| 39 | Male | 63 | Neoplastic Disease | 16h 50min |
| 40 | Male | 57 | Neoplastic Disease | 21h 50min |
| 41 | Male | 61 | Neoplastic Disease | 15h |
| 42 | Female | 49 | Neoplastic Disease | 21h |
| 43 | Male | 65 | Neoplastic Disease | 17h |
| 44 | Male | 44 | Neoplastic Disease | 23h |
| 45 | Female | 58 | Neoplastic Disease | 11h 45min |
| 46 | Female | 57 | Neoplastic Disease | 6h 6min |
| 47 | Male | 55 | Injury/Poisoning | 23h |
| 48 | Male | 64 | Cardiovascular Disease | 22h 30min |
| 49 | Female | 70 | Neoplastic Disease | 22h 25min |
| 50 | Female | 66 | Neoplastic Disease | 16h 25min |

T; total time from the moment of death to the storage of the dissected retinal tissue in a RNA stabilization solution.
