# Supplementary material for: An atlas of gene expression and gene co-regulation in the human retina
Source: Nucleic Acids Res. 2016 May 27;44(12):5773–84. doi: 10.1093/nar/gkw486 (PMC4937338; doi:10.1093/nar/gkw486)
Supplement: SUPPLEMENTARY DATA [file supp_gkw486_nar-00602-z-2016-File014.docx]

# Supplementary Table S3: In-situ hybridization probes

PAQR4

5’-AATTAACCCTCACTAAAGGGCTGCCCATCATCCACTGCAC-3’

5’-TAATACGACTCACTATAGGGGTGGGTGGAAGAGGTCAGTC-3’

ANO2

5’-AATTAACCCTCACTAAAGGGACCTCGTGATGTTCCTGAG-3’

5’-TAATACGACTCACTATAGGGGATGCTGTGCTCCTGACTG-3’

LAPTM4B

5’-AATTAACCCTCACTAAAGGGCGTATGATGatgccactgtg-3’

5’-TAATACGACTCACTATAGGGCAAACTGTCATTGAACTTCAG
