# Supplementary material for: An atlas of gene expression and gene co-regulation in the human retina
Source: Nucleic Acids Res. 2016 May 27;44(12):5773–84. doi: 10.1093/nar/gkw486 (PMC4937338; doi:10.1093/nar/gkw486)
Supplement: SUPPLEMENTARY DATA [file supp_gkw486_nar-00602-z-2016-File016.docx]

# Supplementary Table S5: Observed transcriptome

|  | Genes | Transcripts |
| --- | --- | --- |
| Multiexonic | 19,294 | 71,065 |
| Monoexonic | 4,666 | 6,558 |
| Total | 23,960 | 77,623 |

# Supplementary Table S6: Transcripts with an altered structure compared to GENCODE

First column describes the alteration type. The second column reports the number of transcripts with that alteration. The third column is restricted to the transcripts that are the most expressed isoform in the corresponding gene.

|  | Transcripts | Most-expressed isoform |
| --- | --- | --- |
| All types of alteration | 47,072 (63%) | 11,361 (54%) |
| Altered exon-exon junctions | 39,976 (54%) | 9,204 (43%) |
| Altered 5’ end | 36,954 (50%) | 9,349 (44%) |
| Altered 3’ end | 36,863 (49%) | 8,937 (42%) |
| Altered predicted ORF | 36,759 (49%) | 8,746 (41%) |
| No alteration | 27,462 (37%) | 9,813 (46%) |
| *All transcripts* | *74,534 (100%)* | *21,174 (100%)* |

# Supplementary Table S7: Reference Transcriptome

**TABLE S7 A: Amount of expressed genes and their coding potential, according to Gencode classification.**

|  | All types  (of Gencode) | Protein-coding  (of Gencode) |
| --- | --- | --- |
| Genes | 13792 (24%) | 12035 (65%) |
| Transcripts | 94521 (48%) | 38994 (48%) |

Known genes and transcripts expressed in retina. In parenthesis there is the proportion of the corresponding Gencode entries that are expressed.

**TABLE S7 B: Amount of expressed transcripts and their bio-type, according to Gencode classification.**

| Gencode transcript bio-types | Number | % |
| --- | --- | --- |
| Protein coding | 38994 | 41.29% |
| Processed transcript | 18543 | 19.64% |
| Retained intron | 21962 | 23.26% |
| Nonsense mediated decay | 10427 | 11.04% |
| Processed pseudogene | 65 | 0.07% |
| lincRNA | 1739 | 1.84% |
| Transcribed processed pseudogene | 39 | 0.04% |
| Transcribed unprocessed pseudogene | 159 | 0.17% |
| Unitary pseudogene | 18 | 0.02% |
| Antisense | 2236 | 2.37% |
| Unprocessed pseudogene | 26 | 0.03% |
| Polymorphic pseudogene | 11 | 0.01% |
| Mt rRNA | 2 | 0.00% |
| Sense overlapping | 110 | 0.12% |
| Non-stop decay | 44 | 0.05% |
| Sense intronic | 55 | 0.06% |
| 3-prime overlapping ncrna | 6 | 0.01% |
| TR C gene | 2 | 0.00% |

# Supplementary Table S8: GO classes enriched for most expressed genes (median).

There are no classes significantly depleted for median expression values.

| NAME | SIZE | NES | NOM p-val | FDR q-val |
| --- | --- | --- | --- | --- |
| Biological Process |  |  |  |  |
| Detection of external stimulus | 15 | 1.739 | 0.002 | 0.020* |
| Detection of stimulus | 25 | 1.717 | 0.000 | 0.058 |
| Detection of abiotic stimulus | 16 | 1.708 | 0.001 | 0.067 |
| Response to light stimulus | 39 | 1.659 | 0.000 | 0.349 |
| Response to radiation | 49 | 1.637 | 0.000 | 0.490 |
| Mitochondrial transport | 19 | 1.622 | 0.021 | 0.548 |
| Sensory perception | 122 | 1.611 | 0.000 | 0.566 |
| Nitrogen compound biosynthetic process | 19 | 1.610 | 0.027 | 0.509 |
| DDN damage checkpoint | 18 | 1.591 | 0.033 | 0.597 |
| Response to abiotic stimulus | 70 | 1.591 | 0.000 | 0.539 |
| Cellular component |  |  |  |  |
| Cortical cytoskeleton | 15 | 1.572 | 0.037 | 1.000 |
| Cell cortex part | 18 | 1.572 | 0.039 | 1.000 |
| Cell cortex | 30 | 1.405 | 0.078 | 1.000 |
| Proton transporting two sector ATPase complex | 15 | 1.379 | 0.096 | 1.000 |
| Microtubule | 27 | 1.361 | 0.110 | 1.000 |
| Spindle | 23 | 1.345 | 0.105 | 1.000 |
| Microtubule associated complex | 39 | 1.335 | 0.097 | 1.000 |
| Extracellular space | 85 | 1.324 | 0.080 | 1.000 |
| Trans Golgi network | 16 | 1.308 | 0.147 | 1.000 |
| Cell surface | 41 | 1.286 | 0.130 | 1.000 |
| Molecular function |  |  |  |  |
| RNA dependent ATPase activity | 16 | 1.603 | 0.021 | 1.000 |
| Structural constituent of ribosome | 78 | 1.598 | 0.000 | 1.000 |
| Hydrolyase activity | 19 | 1.582 | 0.031 | 1.000 |
| ATPase activity coupled to transmembrane movement of ions | 16 | 1.570 | 0.038 | 1.000 |
| Carbon oxygen lyase activity | 23 | 1.547 | 0.044 | 1.000 |
| RNA helicase activity | 22 | 1.547 | 0.032 | 0.855 |
| ATPase activity coupled to movement of substances | 28 | 1.519 | 0.044 | 0.919 |
| Hydrolase activity acting on acid anhydrides-catalyzing transmembrane movement of substances | 27 | 1.517 | 0.047 | 0.820 |
| Damaged DNA binding | 17 | 1.510 | 0.053 | 0.767 |
| Primary active transmembrane transporter activity | 28 | 1.502 | 0.039 | 0.731 |

# Supplementary Table S9: Enrichment analysis of different categories of retinal genes

| GROUP OF GENES | SIZE | NES | NOM p-val | FDR q-val | FWER p-val |
| --- | --- | --- | --- | --- | --- |
| ROD PHOTORECEPTORS | 13 | 1.457 | 0.004 | 0.008 | 0.008 |
| ALL PHOTORECEPTORS | 54 | 1.456 | 0.000 | 0.004 | 0.008 |
| RETNET | 214 | 1.385 | 0.000 | 0.020 | 0.059 |
| CONE PHOTORECEPTORS | 18 | 1.239 | 0.094 | 0.072 | 0.262 |

# Supplementary Table S10: GO classes enriched for most variable (high CV) and less variable (low CV) genes

## A: Most variable classes

| NAME | SIZE | NES | NOM p-val | FDR q-val |
| --- | --- | --- | --- | --- |
| **Biological process** |  |  |  |  |
| Vasculature development | 35 | 2.035 | 0.001 | 0.068 |
| Skeletal development | 43 | 2.029 | 0.000 | 0.038 |
| Metal ion transport | 58 | 1.947 | 0.000 | 0.068 |
| Angiogenesis | 29 | 1.939 | 0.000 | 0.056 |
| Response to hypoxia | 24 | 1.918 | 0.002 | 0.054 |
| Anatomical structure formation | 33 | 1.914 | 0.001 | 0.048 |
| Potassium ion transport | 27 | 1.894 | 0.001 | 0.051 |
| Cation transport | 77 | 1.861 | 0.000 | 0.062 |
| Behavior | 42 | 1.854 | 0.000 | 0.061 |
| Monovalent inorganic cation transport | 47 | 1.786 | 0.001 | 0.104 |
| **Cellular component** |  |  |  |  |
| Proteinaceous extracellular matrix | 52 | 2.226 | 0.000 | 0.001 |
| Extracellular matrix | 53 | 2.168 | 0.000 | 0.001 |
| Collagen | 16 | 2.160 | 0.000 | 0.001 |
| Extracellular region part | 135 | 2.152 | 0.000 | 0.001 |
| Extracellular space | 85 | 2.003 | 0.000 | 0.005 |
| Extracellular matrix part | 35 | 1.941 | 0.000 | 0.008 |
| Dendrite | 15 | 1.905 | 0.001 | 0.010 |
| Neuron projection | 17 | 1.834 | 0.001 | 0.022 |
| Voltage gated potassium channel complex | 18 | 1.833 | 0.006 | 0.020 |
| Receptor complex | 26 | 1.784 | 0.004 | 0.029 |
| **Molecular function** |  |  |  |  |
| Cation channel activity | 61 | 2.177 | 0.000 | 0.004 |
| Substrate specific channel activity | 79 | 2.151 | 0.000 | 0.003 |
| Ion channel activity | 77 | 2.143 | 0.000 | 0.002 |
| Gated channel activity | 64 | 2.068 | 0.000 | 0.003 |
| Voltage gated cation channel activity | 37 | 2.025 | 0.000 | 0.006 |
| Metal ion transmembrane transporter activity | 76 | 1.999 | 0.000 | 0.008 |
| Voltage gated channel activity | 42 | 1.972 | 0.001 | 0.010 |
| Structural constituent of cytoskeleton | 38 | 1.958 | 0.000 | 0.010 |
| Potassium channel activity | 21 | 1.887 | 0.003 | 0.025 |
| Integrin binding | 18 | 1.827 | 0.003 | 0.043 |

## B: Less variable

| NAME | SIZE | NES | NOM p-val | FDR q-val |
| --- | --- | --- | --- | --- |
| **Biological process** |  |  |  |  |
| ER to Golgi vesicle mediated transport | 18 | -2.208 | 0.000 | 0.003 |
| Cofactor biosynthetic process | 20 | -1.892 | 0.000 | 0.036 |
| Ribosome biogenesis and assembly | 18 | -1.794 | 0.030 | 0.057 |
| Cofactor metabolic process | 44 | -1.783 | 0.000 | 0.052 |
| Histone modification | 23 | -1.722 | 0.000 | 0.069 |
| Viral reproductive process | 21 | -1.623 | 0.040 | 0.097 |
| Viral infectious cycle | 18 | -1.578 | 0.000 | 0.110 |
| rRNA metabolic process | 15 | -1.531 | 0.046 | 0.127 |
| Covalent chromatin modification | 23 | -1.525 | 0.000 | 0.120 |
| Coenzyme metabolic process | 30 | -1.517 | 0.067 | 0.117 |
| **Cellular component** |  |  |  |  |
| Spliceosome | 51 | -1.607 | 0.000 | 0.178 |
| Nuclear pore | 30 | -1.548 | 0.000 | 0.183 |
| Pore complex | 34 | -1.518 | 0.045 | 0.174 |
| Nucleolar part | 17 | -1.352 | 0.134 | 0.338 |
| Proteasome complex | 23 | -1.206 | 0.175 | 0.575 |
| Small nuclear ribonucleoprotein complex | 22 | -1.187 | 0.226 | 0.557 |
| Mitochondrial lumen | 44 | -1.131 | 0.333 | 0.632 |
| Nuclear membrane | 47 | -1.124 | 0.500 | 0.589 |
| Mitochondrial ribosome | 22 | -1.042 | 0.405 | 0.742 |
| Organellar ribosome | 22 | -0.998 | 0.415 | 0.792 |
| **Molecular function** |  |  |  |  |
| General RNA polymerase ii transcription factor activity | 30 | -1.780 | 0.000 | 0.173 |
| Translation initiation factor activity | 23 | -1.446 | 0.070 | 0.544 |
| Signal sequence binding | 15 | -1.309 | 0.136 | 0.753 |
| Protein N-terminus binding | 32 | -1.177 | 0.345 | 1.000 |
| Single stranded DNA binding | 29 | -1.159 | 0.143 | 0.873 |
| Damaged DNA binding | 17 | -1.090 | 0.354 | 0.964 |
| DNA dependent atpase activity | 18 | -0.977 | 0.478 | 1.000 |
| Phosphoric diester hydrolase activity | 21 | -0.966 | 0.538 | 1.000 |
| Monovalent inorganic cation transmembrane transporter activity | 24 | -0.934 | 0.500 | 1.000 |
| Structure specific DNA binding | 46 | -0.907 | 0.500 | 1.000 |
