# Supplementary material for: An atlas of gene expression and gene co-regulation in the human retina
Source: Nucleic Acids Res. 2016 May 27;44(12):5773–84. doi: 10.1093/nar/gkw486 (PMC4937338; doi:10.1093/nar/gkw486)
Supplement: SUPPLEMENTARY DATA [file supp_gkw486_nar-00602-z-2016-File018.docx]

# Supplementary Table S12

|  | Known genes | Gulty-by-association genes | Guilty and known | Fisher’s p-value | Odds Ratio |
| --- | --- | --- | --- | --- | --- |
| Photoreceptor Genes | 65 | 294 | 39 | < 10-16 | 110 |
| RetNet | 222 | 472 | 56 | < 10-16 | 15 |

Total number of genes 19,295
