# Supplementary material for: An atlas of gene expression and gene co-regulation in the human retina
Source: Nucleic Acids Res. 2016 May 27;44(12):5773–84. doi: 10.1093/nar/gkw486 (PMC4937338; doi:10.1093/nar/gkw486)
Supplement: SUPPLEMENTARY DATA [file supp_gkw486_nar-00602-z-2016-File020.docx]

# Supplementary Text

Description of top candidate genes from Tables S11 and Table S13.

Below, most of the information derived from querying OMIM (http://omim.org/), GeneCard (http://www.genecards.org/), and Mouse Genome Informatics (http://www.informatics.jax.org/). Further references are indicated case by case.

In **bold** are genes that have been validated in vitro.

- AGPAT3 is an ubiquitous acetyltransferase involved in lipid metabolism.
- **ANO2**, known also as TMEM16B, is Ca+2-activated Chloride channel that was demonstrated to be expressed in the mouse photoreceptor synapses (Stöhr et al. 2009). It generates an outward rectifier current that influence the membrane potential and, in turn, the light-dependent firing to second neuron (Lalonde et al.).Our guilty-by-association indicated this gene is significantly co-expressed with known disease-causing genes. We have confirmed that ANO2 is specifically expressed in human photoreceptor cells as predicted by guilty-by-association analysis.
- AIPL1 causes an autosomal recessive retinal diseases (OMIM *604392).
- C2ORF71 causes Retinitis Pigmentosa 34 (OMIM *613425).
- C2orf71 is a kinase with unknown functional activity.
- CPLX4 is a calpain-like protein that is specifically expressed in mouse and rat retina (Reim et al. 2005). Mutation in CPLX4 alters the neurotransmission from photoreceptor to second neurons in mouse models (Reim et al. 2009).
- DPYSL3 participates to the neurite and axonal outgrowth and is expressed during brain development (Nemeroff et al. 1977).
- GNB1 is a guanine nucleotide regulatory protein that activates a cGMP phosphodiesterase in photoreceptor cells (OMIM 139380). There are evidence for its role in a mouse model of retinal disease (Kitamura et al. 2006).
- GPR160 is G-protein coupled receptor with unknown function.
- GSKIP plays a role in photoreceptor cells, since it is a GSK3beta inhibitor (Chou et al. 2006) , which gene influences photoreceptor cells survival via a FGF-mediated mechanism (O’Driscoll et al. 2007).
- GUCA1B causes Retinitis Pigmentosa 48 (OMIM 602275).
- IMPG1 causes Viteliform Macular Dystrophy 4 (OMIM 602870).
- **LAPTM4B** is lysosome-associated transmembrane protein that has been found overexpressed in many cancerous and normal tissue (Kasper et al. 2005). We experimentally confirmed that is expressed in photoreceptor cells.
- LMOD1 is an ocular muscular protein that would be the target of cross-reacting antibodies in the thyroid-associated ophthalmopathy (Dong et al. 1991). According to our guilty-by-association analysis this protein is expressed also in photoreceptor cells.
- IMPG2 causes Viteliform Macular Dystrophy 5 and Retinitis Pigmentosa 56 (OMIM 607056).
- MEF2C is a transcription factor that is involved multiple organ development. Its mutations cause syndromic disease with cerebral malformation (OMIM 600662).
- KCNB1 is a potassium channel which mutations cause a form of heditary epilepsy (OMIM 600397).
- MCUR1 is a mitochondrial Ca+2-importer regulator which knockout disrupts oxidative phosphorylation, lowers cellular ATP and activates AMP kinase-dependent pro-survival autophagy (Mallilankaraman et al. 2012).
- PAIP2B inhibits the translation of mature mRNAs by displacing PABP from the 3’-end (Berlanga et al. 2006). If the involvement of this gene in the retinal pathophysiology will be confirmed, it supports the idea that the retina is low tolerant to dysregulation of RNA-maturation as it was proven with the alterations of splicing machinery (Berger et al. 2010).
- **PAQR4** is a 7-transmembrne domain similar to adiponectin 1 and 2 receptors (ADIPOR1 and ADIPOR2). Both are known to be expressed in photoreceptor cells and the knock-out of the first causing a form of retinitis in mouse (Rice et al. 2015).
- PPP3R1 is a calcium-dependent protein phosphatase that has been found expressed only in testis (OMIM 613821). According to our analysis it is expressed at high level also in the retina and it is co-regulated with genes that are involved in ciliar disfunction and cause Bartel Biedl disease.
- RHO is the visual pigment of the rod cells and it is one the most expressed protein in retina. Its mutations cause retinitis pigmentosa, night vision loss and retinitis punctata albescens (OMIM 180380).
- PRPH2 is a membrane protein involved in photoreceptor disc morphogenesis. Its mutations cause autosomal dominant and recessive retinal diseases (OMIM 179605).
- The SLC24A1 gene encodes a sodium-calcium exchanger expressed in the retina. Its mutations cause an autosomal recessive form of night-blindness (OMIM 603617).
- TMEM136 is a transmembrane protein that is expressed in mouse neuroretina (MGI:2685030).
- WNT mediate many developmental processes during embryogenesis, including anterior-posterior axis formation, cell polarity, and cell migration. During development, retinal myeloid cells produce Wnt ligands to regulate blood vessel branching. Its mutation cause alterations of eye morphology in mouse (OMIM 611514, MGI:1915401).

References

Berger W, Kloeckener-Gruissem B, Neidhardt J. 2010. The molecular basis of human retinal and vitreoretinal diseases. *Prog Retin Eye Res* **29**: 335–375. http://dx.doi.org/10.1016/j.preteyeres.2010.03.004.

Berlanga JJ, Baass A, Sonenberg N. 2006. Regulation of poly(A) binding protein function in translation: Characterization of the Paip2 homolog, Paip2B. *RNA* **12**: 1556–68. http://www.ncbi.nlm.nih.gov/pubmed/16804161.

Chou H-Y, Howng S-L, Cheng T-S, Hsiao Y-L, Lieu A-S, Loh J-K, Hwang S-L, Lin C-C, Hsu C-M, Wang C, et al. 2006. GSKIP is homologous to the Axin GSK3beta interaction domain and functions as a negative regulator of GSK3beta. *Biochemistry* **45**: 11379–89. http://www.ncbi.nlm.nih.gov/pubmed/16981698.

Dong Q, Ludgate M, Vassart G. 1991. Cloning and sequencing of a novel 64-kDa autoantigen recognized by patients with autoimmune thyroid disease. *J Clin Endocrinol Metab* **72**: 1375–81. http://www.ncbi.nlm.nih.gov/pubmed/2026759.

Kasper G, Vogel A, Klaman I, Gröne J, Petersen I, Weber B, Castaños-Vélez E, Staub E, Mennerich D. 2005. The human LAPTM4b transcript is upregulated in various types of solid tumours and seems to play a dual functional role during tumour progression. *Cancer Lett* **224**: 93–103. http://www.ncbi.nlm.nih.gov/pubmed/15911104.

Kitamura E, Danciger M, Yamashita C, Rao NP, Nusinowitz S, Chang B, Farber DB. 2006. Disruption of the gene encoding the beta1-subunit of transducin in the Rd4/+ mouse. *Invest Ophthalmol Vis Sci* **47**: 1293–301. http://www.ncbi.nlm.nih.gov/pubmed/16565360.

Lalonde MR, Kelly ME, Barnes S. Calcium-activated chloride channels in the retina. *Channels (Austin)* **2**: 252–60. http://www.ncbi.nlm.nih.gov/pubmed/18769137.

Mallilankaraman K, Cárdenas C, Doonan PJ, Chandramoorthy HC, Irrinki KM, Golenár T, Csordás G, Madireddi P, Yang J, Müller M, et al. 2012. MCUR1 is an essential component of mitochondrial Ca2+ uptake that regulates cellular metabolism. *Nat Cell Biol* **14**: 1336–43. http://www.ncbi.nlm.nih.gov/pubmed/23178883.

Nemeroff CB, Grant LD, Bissette G, Ervin GN, Harrell LE, Prange AJ. 1977. Growth, endocrinological and behavioral deficits after monosodium L-glutamate in the neonatal rat: possible involvement of arcuate dopamine neuron damage. *Psychoneuroendocrinology* **2**: 179–96. http://www.ncbi.nlm.nih.gov/pubmed/601168.

O’Driscoll C, Wallace D, Cotter TG. 2007. bFGF promotes photoreceptor cell survival in vitro by PKA-mediated inactivation of glycogen synthase kinase 3beta and CREB-dependent Bcl-2 up-regulation. *J Neurochem* **103**: 860–70. http://www.ncbi.nlm.nih.gov/pubmed/17714451.

Reim K, Regus-Leidig H, Ammermüller J, El-Kordi A, Radyushkin K, Ehrenreich H, Brandstätter JH, Brose N. 2009. Aberrant function and structure of retinal ribbon synapses in the absence of complexin 3 and complexin 4. *J Cell Sci* **122**: 1352–61. http://www.ncbi.nlm.nih.gov/pubmed/19386896 (Accessed December 4, 2015).

Reim K, Wegmeyer H, Brandstätter JH, Xue M, Rosenmund C, Dresbach T, Hofmann K, Brose N. 2005. Structurally and functionally unique complexins at retinal ribbon synapses. *J Cell Biol* **169**: 669–80. http://www.ncbi.nlm.nih.gov/pubmed/15911881.

Rice DS, Calandria JM, Gordon WC, Jun B, Zhou Y, Gelfman CM, Li S, Jin M, Knott EJ, Chang B, et al. 2015. Adiponectin receptor 1 conserves docosahexaenoic acid and promotes photoreceptor cell survival. *Nat Commun* **6**: 1–14. http://dx.doi.org/10.1038/ncomms7228.

Stöhr H, Heisig JB, Benz PM, Schöberl S, Milenkovic VM, Strauss O, Aartsen WM, Wijnholds J, Weber BHF, Schulz HL. 2009. TMEM16B, a novel protein with calcium-dependent chloride channel activity, associates with a presynaptic protein complex in photoreceptor terminals. *J Neurosci* **29**: 6809–18. http://www.ncbi.nlm.nih.gov/pubmed/19474308.
